# Modulation of Hepatic Cholesterol-Related Pathways by *Ophiocordyceps sinensis* and Coenzyme Q10 in a Sequential Gut–Liver In Vitro Model

**DOI:** 10.3390/molecules31183265

**Published:** 2026-09-15

**Authors:** Simone Mulè, Rebecca Galla, Matteo Musu, Francesca Parini, Claudio Molinari, Francesca Uberti

**Affiliations:** 1 Department for Sustainable Development and Ecological Transition, University of Piemonte Orientale, Piazza Sant’Eusebio 5, 13100 Vercelli, Italy; simone.mule@uniupo.it (S.M.); 20049586@studenti.uniupo.it (M.M.); claudio.molinari@uniupo.it (C.M.); 2 Noivita s.r.l.s., Spin Off of University of Piemonte Orientale, Strada Privata Curti n. 7, 28100 Novara, Italy; rebecca.galla@noivita.it (R.G.); francescaparini00@gmail.com (F.P.)

**Keywords:** hepatic cholesterol-related pathways, sequential intestinal–hepatic in vitro model, *Ophiocordyceps sinensis*, coenzyme Q10, intestinal transport

## Abstract

Dysregulated hepatic cholesterol handling and oxidative stress represent key targets for nutritional interventions. This study investigated a combination of *Ophiocordyceps sinensis* (formerly *Cordyceps sinensis*) extract and coenzyme Q10 (CoQ10) using a sequential gut–liver in vitro model. Differentiated Caco-2/HT29-MTX co-cultures were used to assess intestinal transport, transepithelial electrical resistance (TEER), and junctional organisation. The resulting basolateral intestinal-conditioned medium was applied to high-glucose-stressed HepG2 cells. The combined treatment-maintained epithelial electrical integrity (TEER > 400 Ω·cm^2^) and tight junction protein abundance, while transiently increasing paracellular fluorescein transport (*Papp*) and enhancing basolateral CoQ10 recovery (+29%). In HepG2 cells, the conditioned medium reduced superoxide production (−38%) and enhanced both DiI-labelled low-density lipoprotein (DiI-LDL) uptake (+26%) and intracellular total bile acid content (+33%). These functional outcomes were accompanied by coordinated changes in protein abundance: a decrease in sterol regulatory element-binding protein 2 (SREBP-2), 3-hydroxy-3-methylglutaryl-CoA reductase (HMGR), and proprotein convertase subtilisin/kexin type 9 (PCSK9) levels, alongside increased low-density lipoprotein receptor (LDLR), cytochrome P450 family 7 subfamily A member 1 (CYP7A1), and ATP-binding cassette transporter A1 (ABCA1) expression. Pharmacological inhibition with dorsomorphin suggested a partial involvement of AMP-activated protein kinase (AMPK) signalling, although this mechanistic assessment remained exploratory. Overall, these findings demonstrate the coordinated modulation of intestinal transport, functional hepatic endpoints, and cholesterol-related protein abundance.

## 1. Introduction

Dysregulated hepatic cholesterol homeostasis is a major contributor to cardiometabolic disease and remains a primary therapeutic target for reducing cardiovascular risk. Although statins effectively lower circulating low-density lipoprotein (LDL) cholesterol and cardiovascular events, their long-term use may be limited by treatment intolerance, variable adherence, and compensatory regulatory responses, including increased oxidative stress and the upregulation of proprotein convertase subtilisin/kexin type 9 (PCSK9), which may attenuate LDL receptor (LDLR)-mediated cholesterol clearance [1,2,3,4,5,6,7]. These limitations have stimulated growing interest in complementary approaches capable of modulating multiple regulatory pathways governing hepatic cholesterol homeostasis rather than acting exclusively through the inhibition of cholesterol synthesis. Alongside their intrinsic biological activity, increasing evidence indicates that intestinal transport and systemic availability are critical determinants of the metabolic efficacy of orally administered bioactive compounds.

Red yeast rice (RYR) has been extensively investigated as a nutraceutical alternative for cholesterol management because of its content of monacolin K, a natural 3-hydroxy-3-methylglutaryl-CoA reductase (HMGR) inhibitor structurally identical to lovastatin [8,9]. However, its clinical application has become increasingly restricted in Europe owing to variability in monacolin content, potential mycotoxin contamination, and the occurrence of statin-like muscle-related adverse effects [10,11]. These concerns have reinforced the search for non-statin-mimetic approaches capable of supporting cholesterol homeostasis through complementary mechanisms while avoiding direct inhibition of HMGR [12,13].

Consequently, increasing attention has been directed towards food-derived bioactive compounds capable of modulating multiple regulatory pathways involved in lipid homeostasis while preserving favourable intestinal transport and downstream metabolic activity [14,15]. Rather than exclusively targeting cholesterol biosynthesis, several emerging nutritional approaches aim to regulate complementary mechanisms regulating hepatic cholesterol handling, including the PCSK9–LDLR axis, ATP-binding cassette transporter A1 (ABCA1)-mediated cholesterol efflux, and cytochrome P450 family 7 subfamily A member 1 (CYP7A1)-dependent bile acid synthesis [16,17,18,19]. These pathways play fundamental roles in maintaining intracellular cholesterol balance and have been extensively investigated in hepatocyte-derived experimental systems. Among these, HepG2 cells represent a well-established in vitro model for studying hepatic lipid metabolism and cholesterol regulation [20,21,22,23].

Among the currently available non-statin-mimetic approaches, several food-derived bioactive compounds have demonstrated complementary effects on lipid metabolism through the modulation of multiple regulatory pathways involved in hepatic cholesterol regulation. Berberine, β-glucans, phytosterols, bergamot-derived polyphenols, omega-3 fatty acids, and probiotics have all been reported to modulate distinct yet interconnected pathways regulating cholesterol metabolism, including LDLR expression, cholesterol absorption, inflammatory signalling, and lipid handling [24,25,26,27]. Within this context, medicinal mushrooms have attracted increasing interest owing to their complex chemical composition and ability to modulate multiple metabolic pathways simultaneously. *Cordyceps* species, including *Cordyceps militaris* and *Ophiocordyceps sinensis* (formerly known as *Cordyceps sinensis*), together with coenzyme Q10 (CoQ10), have emerged as promising candidates for modulating hepatic lipid metabolism, oxidative stress, and cellular bioenergetics.

Among medicinal mushrooms, *Ophiocordyceps sinensis* is a rich source of structurally diverse bioactive compounds with potential relevance to hepatic lipid homeostasis. Its pharmacological activity has been attributed to a complex mixture of polysaccharides, β-glucans, nucleoside derivatives such as cordycepin, and other secondary metabolites whose complementary biological activities contribute to its overall metabolic effects [28,29,30]. Polysaccharide-rich extracts obtained from *Cordyceps* species have been shown to influence cholesterol-related pathways in both in vitro and in vivo experimental models [31,32], while studies performed in hepatocyte-derived systems, including HepG2 cells, have demonstrated the modulation of key regulatory pathways involved in hepatic lipid metabolism, including mechanisms governing LDL receptor availability and cholesterol uptake [33]. In addition to their recognised antioxidant and immunomodulatory properties, β-glucans influence lipid metabolism and cellular adaptation through metabolic signalling pathways, including AMP-activated protein kinase (AMPK)-related mechanisms [30,34]. Likewise, cordycepin has been reported to regulate cellular energy metabolism, attenuate oxidative stress, and modulate signalling pathways associated with hepatic metabolic adaptation, including AMPK-dependent responses [35,36].

CoQ10 is another bioactive compound of considerable interest due to its essential role in mitochondrial electron transport, cellular energy production, and antioxidant defence. Experimental evidence indicates that CoQ10 reduces reactive oxygen species generation, supports mitochondrial function, and contributes to cellular bioenergetic and redox homeostasis through its central role in mitochondrial electron transport. However, despite these favourable biological properties, its high lipophilicity, limited aqueous solubility, and relatively low intestinal absorption may substantially restrict its oral bioavailability and downstream metabolic activity [37,38]. These pharmacokinetic limitations provide a strong rationale for investigating strategies aimed at improving intestinal transport and basolateral availability of CoQ10 following epithelial passage. In this regard, combining CoQ10 with bioactive compounds able to influence intestinal barrier function and transepithelial transport may represent a promising strategy to improve its intestinal recovery while simultaneously targeting complementary mechanisms associated with hepatic cholesterol handling.

Despite increasing evidence supporting the individual biological activities of *Cordyceps*-derived bioactive compounds and CoQ10, important knowledge gaps remain regarding their combined effects on intestinal transport and hepatic cholesterol-related pathways. Most previous investigations have evaluated these compounds independently or have focused exclusively on hepatic responses, without considering the influence of intestinal epithelial passage on the delivery and subsequent biological activity of orally administered bioactive molecules. Because intestinal absorption is a critical determinant of systemic exposure, integrated experimental models recapitulating the sequential intestinal–hepatic in vitro models provide a more physiologically relevant framework for investigating how intestinal transport influences downstream hepatic metabolic responses [39,40,41]. This consideration may be particularly relevant for compounds such as CoQ10, whose limited oral bioavailability is recognised as a principal factor limiting its biological effectiveness. However, previous sequential models have mainly addressed individual compounds [42], while studies on chemicals compounds such as CoQ10 and others have largely examined their direct hepatic effects without accounting for intestinal processing and transport [43,44].

Based on these considerations, the present study investigated the effects of a combination of *Ophiocordyceps sinensis* extract (referred to as *Cordyceps sinensis* sample in Section 2 for consistency) and CoQ10 using a sequential gut–liver in vitro model designed to reproduce intestinal epithelial transfer followed by subsequent hepatic exposure. The primary hypothesis was that intestinal processing of the combined formulation generates a bioaccessible fraction capable of modulating hepatic cholesterol regulation in metabolically stressed HepG2 cells, supporting a functional gut–liver axis. A differentiated Caco-2/HT29-MTX co-culture grown on Transwell^®^ inserts was employed to evaluate the preservation of intestinal barrier junctional organisation, the maintenance of baseline electrical integrity, transepithelial transport, and basolateral recovery of bioactive compounds, while the resulting basolateral intestinal-conditioned medium, representing the bioaccessible fraction of the treatments, was subsequently used to treat HepG2 cells to assess oxidative stress and the modulation of key pathways involved in hepatic cholesterol regulation with a further AMPK signalling analysis investigated as an exploratory mechanistic pathway. This sequential in vitro approach avoids direct exposure of hepatocytes to the nominal apical doses and more closely reflects the physiological sequence of intestinal absorption followed by hepatic processing.

Emphasis was placed on the coordinated regulation of hepatic cholesterol regulation by evaluating pathways involved in synthesis, uptake, efflux, and bile acid metabolism, including HMGR, LDLR, PCSK9, ABCA1, CYP7A1, and sterol regulatory element-binding protein 2 (SREBP-2). In parallel, the chemical characterisation of the *Ophiocordyceps sinensis* extract was performed to support the interpretation of the biological findings and relate its phytochemical composition to the observed biological activity. Finally, the biological responses were compared to those obtained using an RYRF-based reference comparator serving as a commercially relevant nutraceutical reference comparator rather than a pharmacological statin surrogate. This provided a benchmark against an established cholesterol-lowering nutraceutical, enabling the comparative evaluation of intestinal transport and hepatic cholesterol regulatory activity within the same experimental framework.

## 2. Results

### 2.1. Chemical Characterisation of Fungal Extract

Preliminary chemical characterisation of the *Ophiocordyceps sinensis* extract (hereafter referred to as *Cordyceps sinensis* throughout the Results, figures and tables) was performed prior to the biological experiments to characterise its principal constituents. The analytical results are summarised in Table 1. Total carbohydrate content, determined by the phenol–sulfuric acid assay, was 53.2 ± 2.3% (*w*/*w*). β-glucan content was assessed using two complementary analytical methods. The Congo red assay measured the total β-1,3/1,6-glucan fraction, yielding 43.7 ± 1.5% (*w*/*w*). Analysis with the Aniline Blue assay showed a β-1,3-glucan content of 31.8 ± 1.3% (*w*/*w*). Reverse-phase high-performance liquid chromatography coupled with ultraviolet detection (RP-HPLC-UV) analysis identified and quantified cordycepin in the extract at 0.47 ± 0.05% (*w*/*w*).

### 2.2. Dose–Response Evaluation of Cordyceps sinensis, CoQ10, and RYRF in Caco-2/HT29-MTX Co-Cultures

**Figure 1:**
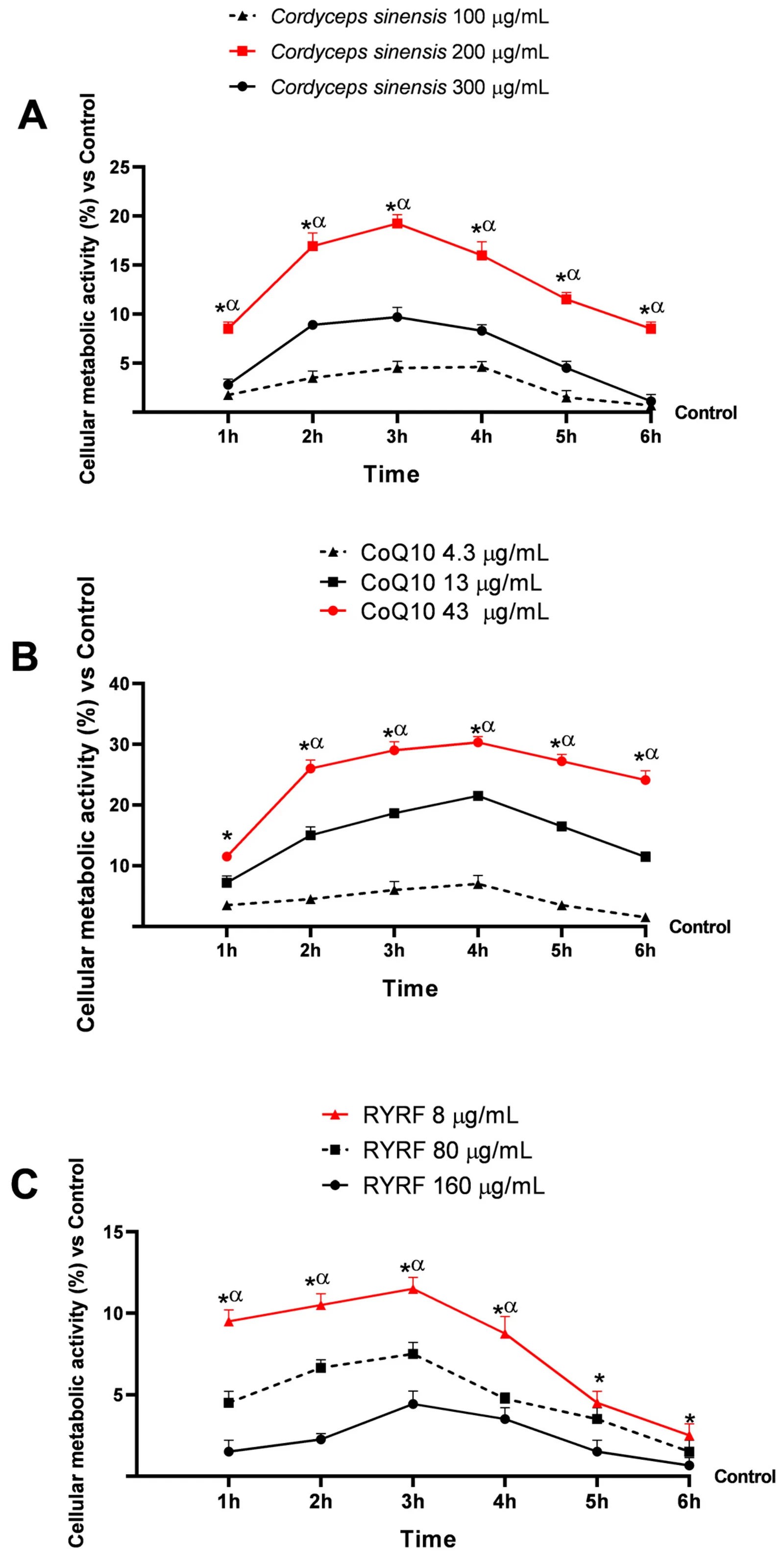
Dose- and time-dependent effects of *Cordyceps sinensis*, CoQ10, and RYRF on the cellular metabolic activity of differentiated Caco-2/HT29-MTX co-cultures following 1–6 h exposure. (**A**) *Cordyceps sinensis*; (**B**) CoQ10; (**C**) RYRF. Cellular metabolic activity was determined using the MTT assay. Data are expressed as mean ± SD from five independent biological experiments performed in technical triplicate and are presented as percentage change relative to the untreated control. Statistical significance was determined by one-way ANOVA followed by Dunnett’s post hoc test. * *p* < 0.05 vs. untreated control; α *p* < 0.05 vs. the other tested concentrations.

Dose–response experiments were performed in differentiated Caco-2/HT29-MTX co-cultures to identify the evaluate cellular metabolic activity and establish cytocompatibility concentration ranges for the subsequent intestinal and sequential gut–liver experiments. Cells were exposed to three concentrations of each test compound for 1–6 h, and cellular metabolic activity was evaluated as mitochondrial reducing capacity and was evaluated using the MTT assay (Figure 1). These experiments were not intended to identify optimal therapeutic doses, but rather to exclude concentrations associated with impaired metabolic activity and select treatment conditions compatible with subsequent transport and mechanistic studies.

As shown in Figure 1A, *Cordyceps sinensis* exhibited a bell-shaped metabolic profile, with the highest metabolic activity observed at 200 μg/mL, whereas mitochondrial reducing capacity declined at the highest concentration tested (*p* < 0.05). CoQ10 (Figure 1B) showed a progressive increase in metabolic activity across the tested concentration range, with 43 μg/mL producing the highest value (*p* < 0.05). In contrast, the reference RYRF-based nutraceutical comparator (Figure 1C) displayed an inverse dose–response profile, with 8 μg/mL producing higher cell viability than the two higher concentrations (*p* < 0.05). Based on these findings, and with the aim of selecting the highest concentrations maintaining normal cellular metabolic activity without inducing cytotoxicity, 200 μg/mL for *Cordyceps sinensis*, 43 μg/mL for CoQ10, and 8 μg/mL for RYRF were selected for all subsequent experiments. The combined treatment was therefore performed using *Cordyceps sinensis* (200 μg/mL) and CoQ10 (43 μg/mL).

### 2.3. Effects of Cordyceps sinensis and CoQ10 on Intestinal Epithelial Electrical Integrity, Junctional Organisation, and Paracellular Transport Properties

**Figure 2:**
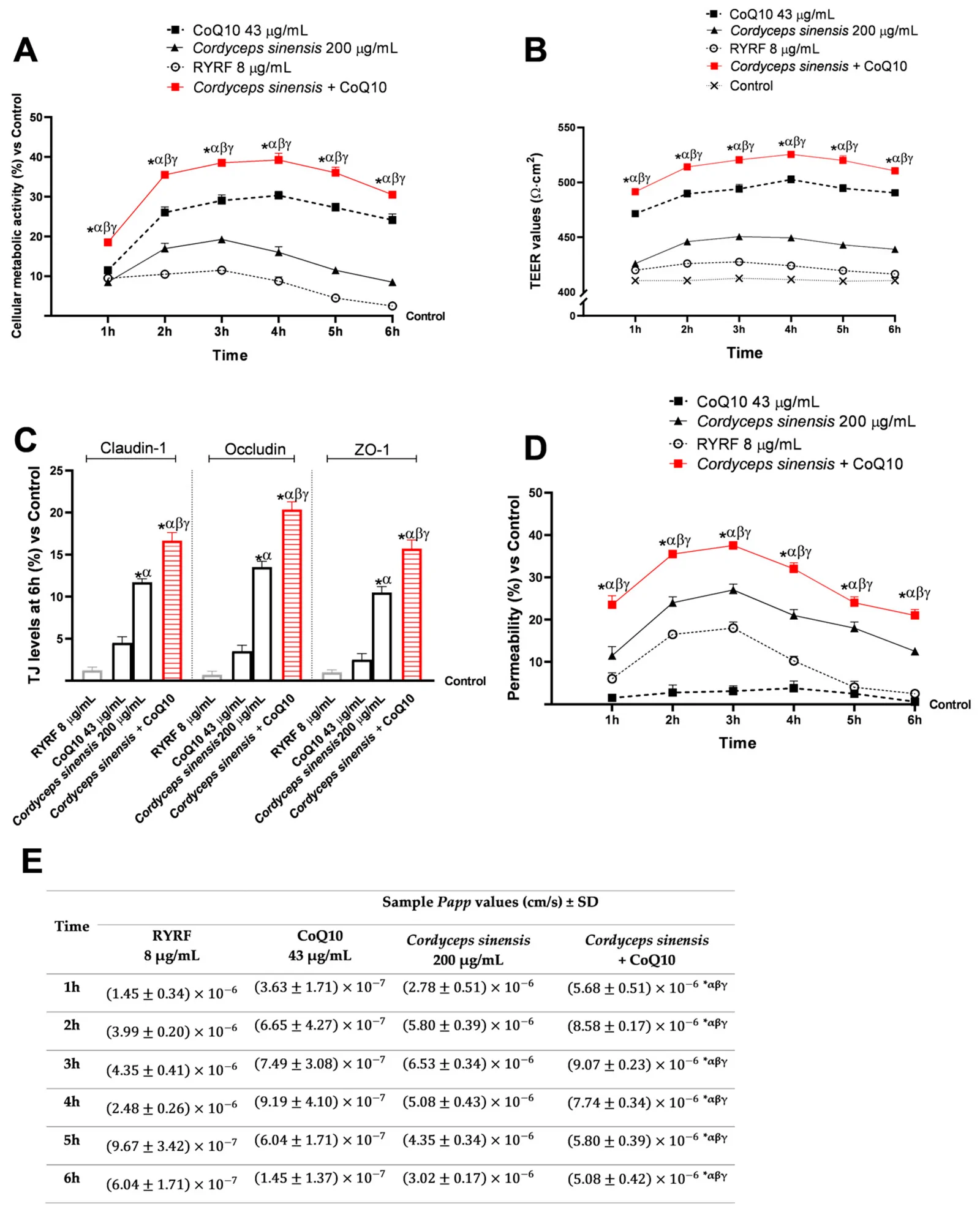
Effects of *Cordyceps sinensis*, CoQ10, and their combination on intestinal responses in differentiated Caco-2/HT29-MTX co-cultures following 1–6 h exposure. (**A**) Cellular metabolic activity determined by the MTT assay. (**B**) TEER values as a measure of electrical barrier properties. (**C**) Tight junction protein expression (Claudin-1, Occludin, and ZO-1) determined by ELISA. (**D**) Paracellular permeability evaluated by fluorescein transfer across the intestinal monolayer. (**E**) *Papp* value for each sample, providing a quantitative assessment of paracellular transport. Data are expressed as mean ± SD from five independent biological experiments performed in technical triplicate. Data in panels (**A**), (**C**) and (**D**) are presented as percentage change relative to the untreated control, whereas panel E reports absolute values expressed as cm/s. Statistical significance was assessed using two-way repeated-measures ANOVA followed by Tukey’s post hoc test for panels (**A**), (**B**), (**D**), (**E**) and by one-way ANOVA followed by Dunnett’s post hoc test for panel C. Exact *p*-values are reported in the text. * *p* < 0.05 vs. untreated control; α *p* < 0.05 vs. RYRF nutraceutical comparator (8 μg/mL); β *p* < 0.05 vs. CoQ10 (43 μg/mL); γ *p* < 0.05 vs. *Cordyceps sinensis* (200 μg/mL).

Following the selection of the experimental concentrations, differentiated Caco-2/HT29-MTX co-cultures grown on Transwell^®^ inserts were used to evaluate cell viability, electrical barrier properties, tight junction protein expression, paracellular transport, and the transepithelial transfer of the test compounds after 1–6 h of exposure (Figure 2).

Cellular metabolic activity increased across all experimental conditions (Figure 2A), with the combination of *Cordyceps sinensis* and CoQ10 producing significantly higher data than either treatment alone throughout the experimental time course (*p* < 0.001). The greatest increase was observed between 3 and 4 h, corresponding to approximately 39% above the untreated control.

Baseline electrical integrity was assessed by transepithelial electrical resistance (TEER) measurements (Figure 2B). TEER values remained above the predefined acceptance threshold (400 Ω·cm^2^) throughout the study in all experimental groups. Among the individual treatments, CoQ10 (43 μg/mL) showed the highest TEER values, while the combined treatment resulted in the greatest increase, reaching approximately 525 Ω·cm^2^ after 3–4 h of exposure (*p* < 0.001). Detailed TEER measurements at each experimental time point are reported in Appendix B.

Analysis of tight junction proteins after 6 h of treatment further supported the TEER findings (Figure 2C). Claudin-1, Occludin, and Zonula occludens-1 (ZO-1) expression increased in all treated groups compared to the untreated control. Among the individual treatments, *Cordyceps sinensis* (200 μg/mL) induced the greatest increase in tight junction protein expression, whereas the combined treatment produced significantly higher levels than either individual treatment or RYRF (8 μg/mL) (*p* < 0.001 vs. CoQ10 and RYRF; *p* = 0.005 vs. *Cordyceps sinensis*).

Paracellular permeability was subsequently evaluated by measuring fluorescein transfer across the intestinal monolayer (Figure 2D). Among the individual treatments, *Cordyceps sinensis* (200 μg/mL) produced a greater increase in fluorescein transfer than CoQ10 (43 μg/mL), with values comparable to those observed with RYRF (8 μg/mL) during the first 4 h of incubation. The combined treatment produced the greatest increase in fluorescein transfer throughout the experimental period (*p* < 0.001), reaching a maximum increase of approximately 37.5% relative to the untreated control after 3 h. TEER values remained above the predefined acceptance threshold throughout the experimental period in all experimental groups. To provide an absolute quantitative assessment of this paracellular transport, the apparent permeability coefficient (*Papp*) was calculated (Figure 2E). In agreement with the percentage change flux data, the *Papp* values significantly increased following exposure to the combined treatment compared to both the untreated control and individual agents (*p* < 0.001), indicating a selective and transient increase in paracellular permeability.

To confirm transepithelial passage of the tested compounds, cordycepin and CoQ10 were quantified in the basolateral compartment by RP-HPLC-UV after 6 h of apical exposure. As summarised in Table A1 (Appendix A), both compounds were detected following the administration of the individual treatments, corresponding to basolateral recoveries of 28.7% (0.27 μg/mL) for cordycepin and 2.6% (1.12 μg/mL) for CoQ10 (*p* < 0.05). Administration of the combined treatment increased basolateral recovery to 36.2% (0.34 μg/mL) for cordycepin and 3.7% (1.58 μg/mL) for CoQ10, representing increases of approximately 20% and 29%, respectively, compared to the corresponding individual treatments (*p* = 0.012 vs. individual treatment). Cordycepin and CoQ10 were the only bioactive constituents directly quantified in the basolateral compartment (RP-HPLC-UV analysis). A comprehensive mass-balance analysis was further performed to evaluate the recovery and distribution of cordycepin and CoQ10 across the Transwell^®^ system, including potential losses due to adsorption, precipitation, or cellular retention (Appendix A, Table A2). Cordycepin showed high overall recovery (>90%), whereas CoQ10 recovery was lower when administered alone (68.2%) but increased to 85.8% when combined with Ophiocordyceps sinensis, reducing the unaccounted fraction from ~32% to ~14%.

### 2.4. HepG2 Cell Responses Following Simulated Intestinal Passage

**Figure 3:**
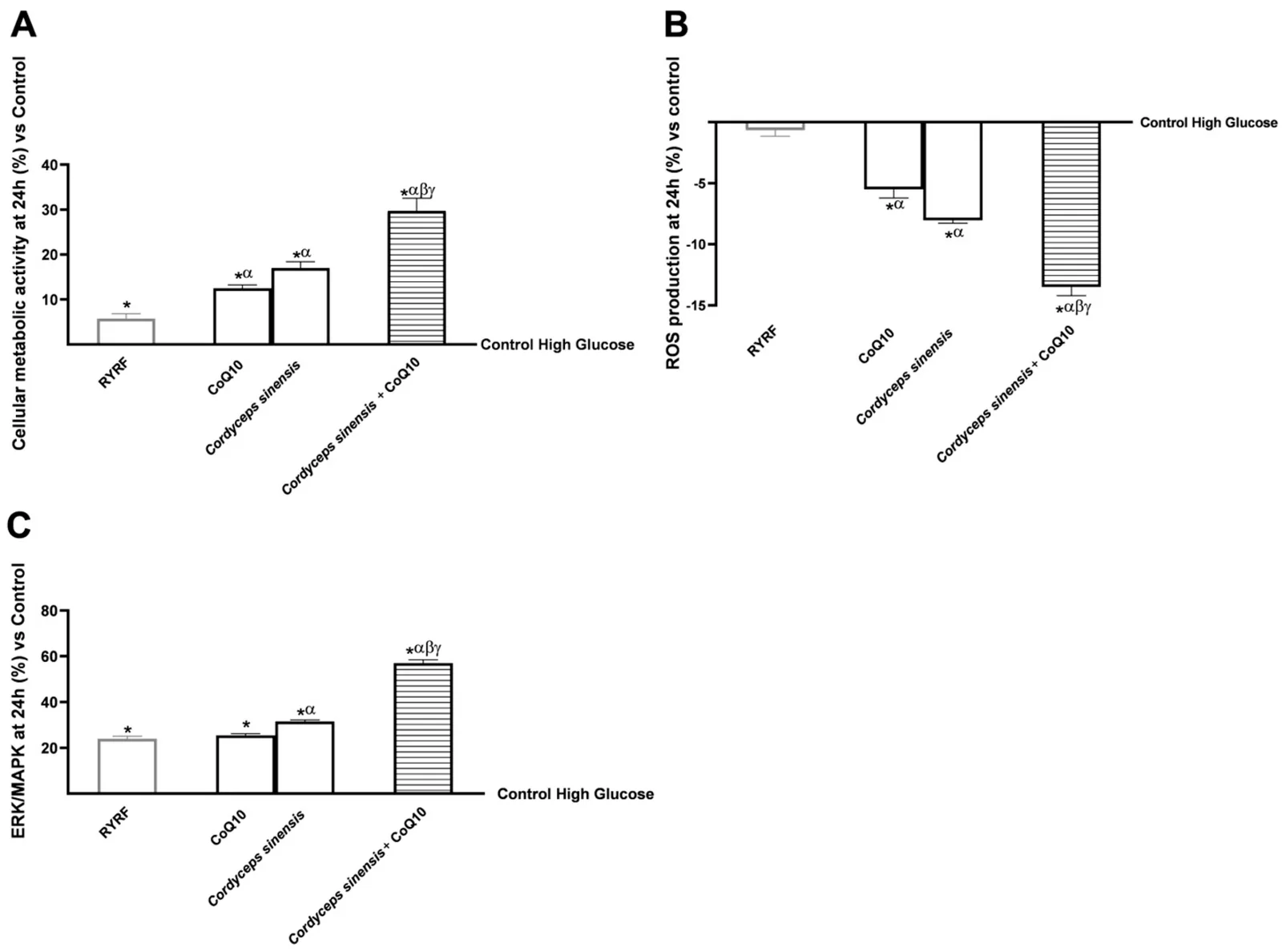
HepG2 cell responses following exposure to basolateral intestinal-conditioned medium collected from the intestinal co-culture model after apical treatment with the tested compounds. Cells were maintained under high-glucose conditions (30 mM D-glucose) and exposed for 24 h. (**A**) Cellular metabolic activity determined by the MTT assay. (**B**) Superoxide production determined using the cytochrome c reduction assay. (**C**) ERK/MAPK activation determined using a specific ELISA kit. Data are expressed as mean ± SD from five independent biological experiments performed in technical triplicate and are presented as percentage change relative to the untreated high-glucose control (0% reference). Statistical significance was determined by one-way ANOVA followed by Dunnett’s post hoc test. Exact *p*-values are reported in the text. * *p* < 0.05 vs. High-glucose control α *p* < 0.05 vs. RYRF-derived basolateral fraction (nutraceutical comparator; obtained from 8 μg/mL nominal apical treatment); β *p* < 0.05 vs. CoQ10 basolateral fraction (1.12 μg/mL); γ *p* < 0.05 vs. *Cordyceps sinensis* basolateral fraction (containing 0.27 μg/mL cordycepin).

To evaluate the hepatic responses following intestinal passage, HepG2 cells were exposed for 24 h to basolateral intestinal-conditioned medium collected from the differentiated Caco-2/HT29-MTX intestinal model after apical treatment with the tested compounds (Figure 3). Therefore, the study followed epithelial processing and transepithelial passage, rather than at predefined nominal concentrations of the original formulations. These basolateral fractions therefore represented the intestinal-processed material available for subsequent hepatic exposure. Hepatic exposure is described based on the experimentally quantified intestinal basolateral concentrations of cordycepin and CoQ10 (Table A1): 0.27 μg/mL cordycepin in the *Cordyceps sinensis* alone group, 1.12 μg/mL CoQ10 in the CoQ10 alone group, and 0.34 μg/mL cordycepin and 1.58 μg/mL CoQ10 in the combined treatment group. Dose–response assessments (Section 2.3) were performed in the apical intestinal compartment to identify cytocompatible concentrations prior to generation of the basolateral fractions.

Hepatic cellular metabolic activity, superoxide production, and extracellular signal-regulated kinase 1/2 (ERK1/2) related to mitogen-activated protein kinase (MAPK) activation (reported as ERK/MAPK) were subsequently assessed. HepG2 cells were maintained under high-glucose conditions (30 mM D-glucose) to establish a metabolically stressed environment. A physiological glucose condition (5 mM D-glucose) and an osmotic control (5 mM D-glucose + 24.5 mM D-mannitol) were included to define baseline hepatic responses and exclude non-specific hyperosmolar effects. As shown in Supplementary Table S1, metabolic responses did not differ significantly between the two controls, indicating that the alterations observed under the high-glucose control were attributable to glucose-induced metabolic stress rather than osmolarity. All results were normalised to untreated cells cultured under the same high-glucose conditions (high-glucose control; 0% reference).

Cellular metabolic activity increased in all treatment groups compared to the untreated high-glucose control (Figure 3A). The basolateral fraction containing CoQ10 (1.12 μg/mL) and that containing cordycepin (0.27 μg/mL) from *Cordyceps sinensis* produced significantly greater increases than the nutraceutical comparator RYRF (*p* = 0.002). The combined treatment (containing 0.34 μg/mL cordycepin and 1.58 μg/mL CoQ10) further increased cellular metabolic activity compared with either individual treatment (*p* < 0.001).

Superoxide production, determined by the cytochrome c reduction assay, decreased in all treatment groups relative to the untreated high-glucose control (Figure 3B), reaching statistical significance for all treatments except RYRF-derived fraction. Among the individual treatments, the basolateral fraction containing CoQ10 (1.12 μg/mL) and that containing cordycepin (0.27 μg/mL) from *Cordyceps sinensis* produced significantly greater reductions than RYRF-derived fraction (*p* < 0.05). The combined treatment (containing 0.34 μg/mL cordycepin and 1.58 μg/mL CoQ10) resulted in the lowest superoxide levels and produced a significantly greater reduction than either individual treatment (*p* < 0.001).

ERK/MAPK activation was subsequently evaluated as an intracellular signalling response (Figure 3C). The basolateral fraction containing CoQ10 (1.12 μg/mL) produced ERK/MAPK activation comparable to that observed with RYRF (8 μg/mL), whereas basolateral fraction with cordycepin (0.27 μg/mL) from *Cordyceps sinensis* induced significantly greater activation (*p* < 0.05). The combined treatment containing 0.34 μg/mL cordycepin and 1.58 μg/mL CoQ10) produced the highest ERK/MAPK activation, significantly exceeding those of both individual treatments and the nutraceutical comparator-derived fraction (*p* < 0.001). Quantitatively, ERK/MAPK activation increased by approximately 56% relative to the RYRF-derived fraction and the CoQ10 basolateral fraction (1.12 μg/mL), and by 45% relative to the cordycepin basolateral fraction (0.27 μg/mL) from *Cordyceps sinensis*.

### 2.5. Effects of Cordyceps sinensis and CoQ10 on Cholesterol Synthesis, Content, and LDL Uptake

**Figure 4:**
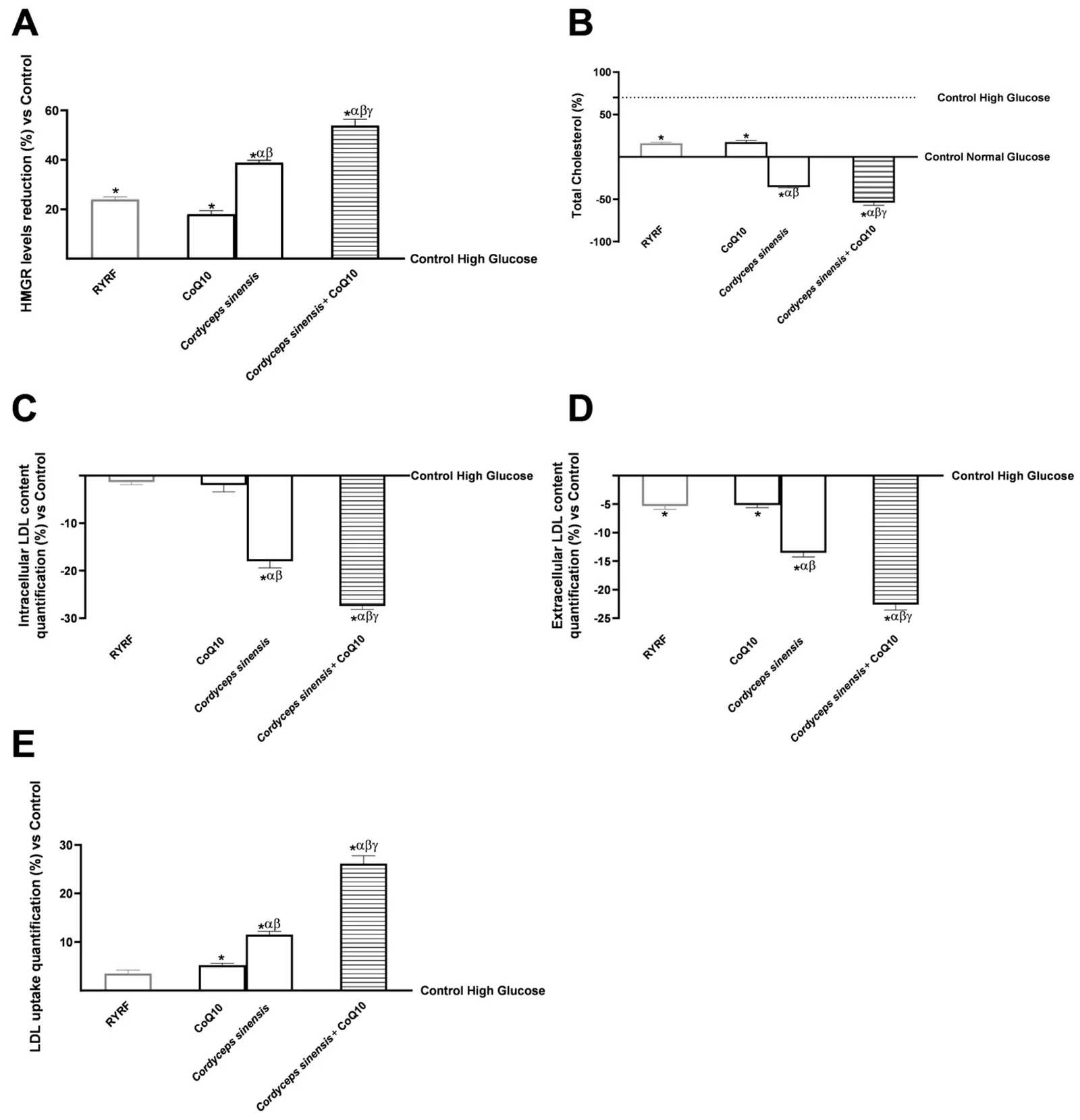
Effects of the tested treatments on hepatic cholesterol-related pathways in HepG2 cells maintained under high-glucose conditions following exposure to basolateral supernatants from the intestinal co-culture model. (**A**) HMGR levels determined by ELISA. (**B**) Total intracellular cholesterol measured using the Cholesterol Quantitation Kit. (**C**) Intracellular LDL-associated cholesterol determined by a colourimetric assay. (**D**) Residual extracellular LDL-associated cholesterol determined by a colourimetric assay. (**E**) LDL uptake assessed using the DiI-LDL uptake assay. Data are expressed as mean ± SD from five independent biological experiments performed in technical triplicate and are presented as percentage change relative to the untreated high-glucose control (0% reference). Statistical significance was determined by one-way ANOVA followed by Dunnett’s post hoc test. Exact *p*-values are reported in the text. * *p* < 0.05 vs. high-glucose control; α *p* < 0.05 vs. RYRF-derived basolateral fraction (nutraceutical comparator; obtained from 8 μg/mL nominal apical treatment); β *p* < 0.05 vs. CoQ10 basolateral fraction (1.12 μg/mL); γ *p* < 0.05 vs. *Cordyceps sinensis* basolateral fraction (containing 0.27 μg/mL cordycepin).

To further investigate hepatic cholesterol-related pathways following simulated intestinal passage, HepG2 cells maintained under high-glucose conditions were exposed for 24 h to basolateral supernatants collected from the intestinal co-culture model. The effects of the treatments on cholesterol metabolism were evaluated by measuring HMGR, total intracellular cholesterol, intracellular LDL-associated cholesterol, residual extracellular LDL-associated cholesterol, and LDL uptake (Figure 4).

HMGR levels were reduced in all treatment groups compared to the untreated high-glucose control (Figure 4A). Among the individual treatments, the basolateral fraction containing cordycepin (0.27 μg/mL) from *Cordyceps sinensis* produced the greatest reduction (*p* < 0.05), whereas the basolateral fraction containing CoQ10 (1.12 μg/mL) and RYRF-derived fraction showed comparable effects. The combined treatment (containing 0.34 μg/mL cordycepin and 1.58 μg/mL CoQ10) produced the largest decrease in HMGR levels, significantly exceeding both the individual treatments and the nutraceutical comparator-derived fraction (*p* < 0.001 vs. RYRF and CoQ10; *p* = 0.002 vs. *Cordyceps sinensis*). Quantitatively, HMGR levels were reduced by approximately 54% relative to the untreated control, 59% relative to RYRF-derived fraction and basolateral fraction containing CoQ10 (1.12 μg/mL), and 28% relative to the basolateral fraction containing cordycepin (0.27 μg/mL) from *Cordyceps sinensis*.

A comparable pattern was observed for total intracellular cholesterol (Figure 4B). All treatments significantly reduced intracellular cholesterol compared to the untreated high-glucose control (*p* < 0.05). Among the individual treatments, the basolateral fraction containing cordycepin (0.27 μg/mL) from *Cordyceps sinensis* produced the greatest reduction, whereas the combined treatment resulted in the lowest intracellular cholesterol levels among all experimental groups (*p* < 0.001 vs. RYRF and CoQ10; *p* = 0.002 vs. *Cordyceps sinensis*). Relative to the untreated control, total intracellular cholesterol decreased by approximately 57%, whereas reductions of 68.5% and 31% were observed relative to RYRF-derived fraction, basolateral fraction containing CoQ10 (1.12 μg/mL) and the basolateral fraction containing cordycepin (0.27 μg/mL) from *Cordyceps sinensis*, respectively. Exploratory Bliss independence analysis (Appendix A, Table A3) indicated that the combined response exceeded the predicted additive effect, suggesting a greater-than-additive mathematical interaction under the specific concentration combination tested. Raw total cholesterol values are reported in Appendix B, corresponding to the data presented in Figure 4B.

Intracellular LDL-associated cholesterol was also significantly affected by the treatments (Figure 4C). Among the individual treatments, the basolateral fraction containing cordycepin (0.27 μg/mL) from *Cordyceps sinensis* produced the greatest reduction (*p* < 0.05), whereas basolateral fraction containing CoQ10 (1.12 μg/mL) showed values comparable to those observed with RYRF (8 μg/mL). The combined treatment (containing 0.34 μg/mL cordycepin and 1.58 μg/mL CoQ10) further reduced intracellular LDL-associated cholesterol compared to all individual treatments (*p* < 0.001 vs. RYRF and CoQ10; *p* = 0.003 vs. *Cordyceps sinensis*), corresponding to reductions of approximately 27.5% relative to the untreated high-glucose control, 33% relative to the basolateral fraction containing cordycepin (0.27 μg/mL) from *Cordyceps sinensis*, and significantly lower values than those observed with both basolateral fraction related to CoQ10 and RYRF.

Residual extracellular LDL-associated cholesterol also decreased in response to all treatments (Figure 4D). All treatment groups showed significantly lower residual extracellular LDL-associated cholesterol than the untreated high-glucose control (*p* < 0.05). Among the individual treatments, the basolateral fraction containing cordycepin (0.27 μg/mL) from *Cordyceps sinensis* produced the greatest reduction, whereas basolateral fraction containing CoQ10 (1.12 μg/mL) showed values comparable to those of RYRF-derived fraction. The combined treatment (containing 0.34 μg/mL cordycepin and 1.58 μg/mL CoQ10) resulted in the lowest residual extracellular LDL-associated cholesterol, significantly lower than that observed with each individual treatment and the nutraceutical comparator-derived fraction (*p* < 0.001). Quantitatively, residual extracellular LDL-associated cholesterol decreased by approximately 23% relative to the untreated high-glucose control, 76% relative to RYRF-derived fraction and basolateral fraction containing CoQ10 (1.12 μg/mL), and 40% relative to basolateral fraction containing cordycepin (0.27 μg/mL) from *Cordyceps sinensis*.

LDL uptake was subsequently evaluated using the DiI-LDL uptake assay (Figure 4E). RYRF-derived fraction produced only a modest increase in LDL uptake, whereas basolateral fraction containing CoQ10 (1.12 μg/mL) induced a slightly greater response. The basolateral fraction containing cordycepin (0.27 μg/mL) from *Cordyceps sinensis* significantly increased LDL uptake by approximately 11.3% relative to the untreated high-glucose control (*p* < 0.001). The combined treatment (containing 0.34 μg/mL cordycepin and 1.58 μg/mL CoQ10) produced the greatest increase in LDL uptake, approximately 26.1% above the untreated high-glucose control (*p* < 0.001), and significantly exceeded the responses observed with either individual treatment (*p* < 0.001). Relative to basolateral fractions related to RYRF and CoQ10, LDL uptake increased by approximately 87%, whereas an increase of 56% was observed relative to basolateral fraction containing cordycepin (0.27 μg/mL) from *Cordyceps sinensis*. Raw DiI-LDL uptake data are reported in Appendix B, corresponding to the data presented in Figure 4E. DiI-LDL uptake was further assessed under LPDS conditions in the presence of a 50-fold excess of unlabelled LDL. As shown in Supplementary Figure S1 and summarised in Table S2, the increased DiI-LDL fluorescence following treatment predominantly reflected specific receptor-mediated internalisation (Supplementary Figure S2).

### 2.6. Effects of Cordyceps sinensis and CoQ10 on Cholesterol Catabolism and Efflux-Related Pathways

**Figure 5:**
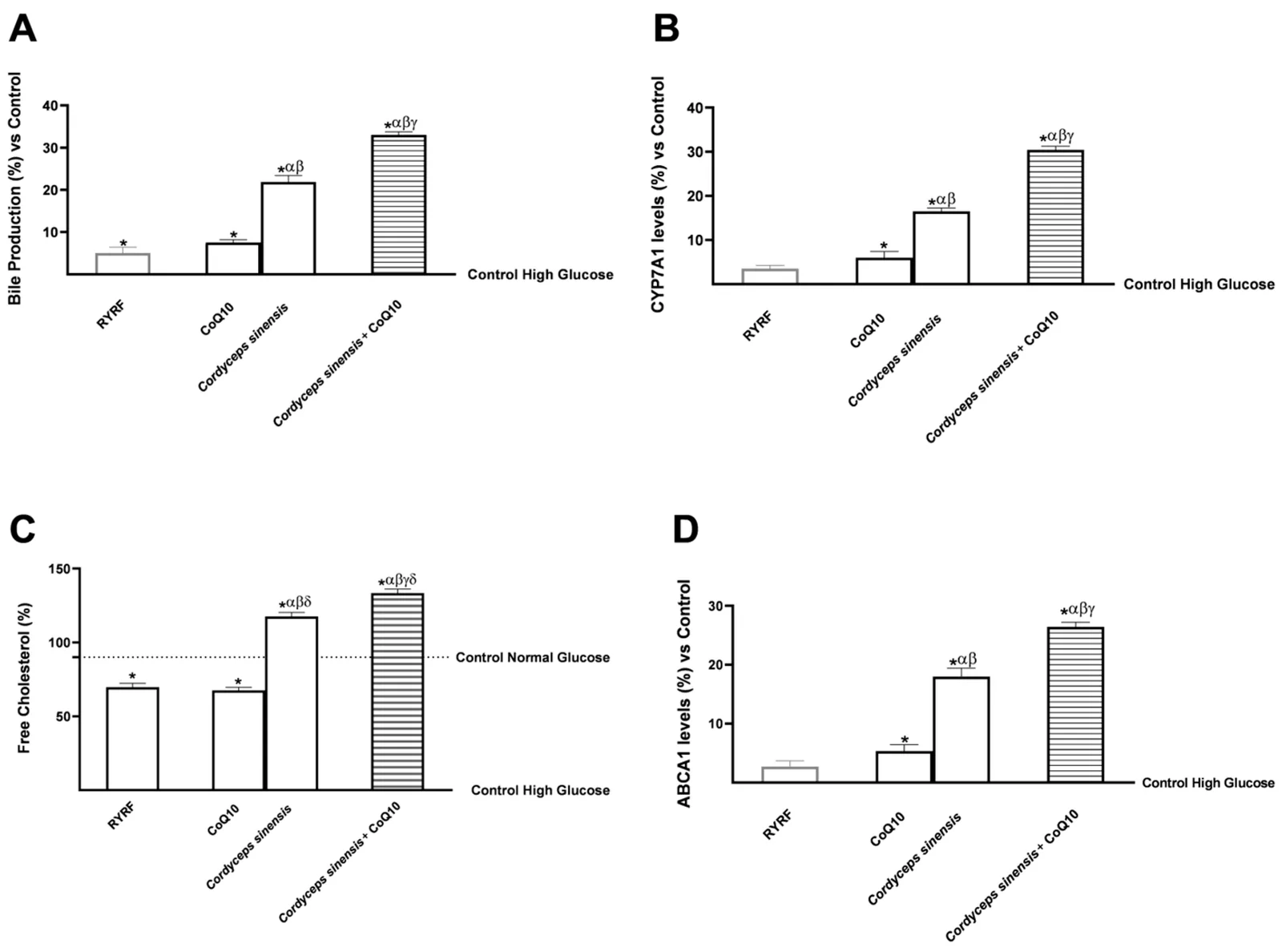
Effects of the tested treatments on hepatic cholesterol catabolism and efflux-related pathways in HepG2 cells maintained under high-glucose conditions following exposure to basolateral supernatants from the intestinal co-culture model. (**A**) Intracellular total bile acid content determined using a fluorometric total bile acid assay. (**B**) CYP7A1 protein levels determined by ELISA. (**C**) Intracellular free cholesterol measured using the Cholesterol Quantitation Kit. (**D**) ABCA1 protein levels determined by ELISA. The untreated high-glucose control (30 mM D-glucose) is shown as the 0% reference line. The dotted line represents the normal-glucose control (5 mM D-glucose). Data are expressed as mean ± SD from five independent biological experiments performed in technical triplicate and are presented as percentage change relative to the untreated high-glucose control. Statistical significance was determined by one-way ANOVA followed by Dunnett’s post hoc test. Exact *p*-values are reported in the text. * *p* < 0.05 vs. high-glucose control; α *p* < 0.05 vs. RYRF-derived basolateral fraction (nutraceutical comparator; obtained from 8 μg/mL nominal apical treatment); β *p* < 0.05 vs. CoQ10 basolateral fraction (1.12 μg/mL); γ *p* < 0.05 vs. *Cordyceps sinensis* basolateral fraction (containing 0.27 μg/mL cordycepin); δ *p* < 0.05 vs. normal-glucose control.

Following the effects observed on cholesterol synthesis and LDL metabolism, additional analyses were performed to evaluate pathways associated with hepatic cholesterol catabolism and cellular cholesterol handling. Specifically, intracellular total bile acid content, CYP7A1 protein levels, intracellular free cholesterol, and ABCA1 protein levels were assessed after HepG2 cells were exposed to basolateral supernatants from the intestinal co-culture model (Figure 5).

Intracellular total bile acid content increased significantly in all treatment groups compared to the untreated high-glucose control (Figure 5A; *p* < 0.05). Among the individual treatments, basolateral fraction containing cordycepin (0.27 μg/mL) from *Cordyceps sinensis* produced the greatest increase, whereas basolateral fraction containing CoQ10 (1.12 μg/mL) and RYRF-derived fraction (8 μg/mL) showed comparable responses. The combined treatment (containing 0.34 μg/mL cordycepin and 1.58 μg/mL CoQ10) resulted in the highest intracellular total bile acid levels, significantly exceeding those of both individual treatments and the nutraceutical comparator-derived fraction (*p* < 0.001 vs. RYRF and CoQ10; *p* = 0.002 vs. *Cordyceps sinensis*). Quantitatively, intracellular total bile acid content increased by approximately 33% relative to the untreated high-glucose control, 81% relative to basolateral fraction related to RYRF and CoQ10, and 34% relative to basolateral fraction containing cordycepin (0.27 μg/mL) from *Cordyceps sinensis*. Exploratory Bliss independence analysis (Appendix A, Table A4) indicated that the combined response exceeded the predicted additive effect, consistent with a greater-than-additive mathematical interaction. Raw data of intracellular total bile acid levels are reported in Appendix B, corresponding to the data presented in Figure 5A.

CYP7A1 protein levels were subsequently determined by ELISA (Figure 5B). RYRF (8 μg/mL) produced only a modest increase in CYP7A1 protein levels, whereas basolateral fraction containing CoQ10 (1.12 μg/mL) and that containing cordycepin (0.27 μg/mL) from *Cordyceps sinensis* (induced significantly higher levels than the untreated high-glucose control (*p* < 0.05). The combined treatment (containing 0.34 μg/mL cordycepin and 1.58 μg/mL CoQ10) produced the greatest increase in CYP7A1 protein levels, corresponding to approximately 30.4% above the untreated high-glucose control and significantly exceeding all individual treatments (*p* < 0.001).

Intracellular free cholesterol was then quantified (Figure 5C). Compared to the untreated high-glucose control, all treatments significantly increased intracellular free cholesterol (*p* < 0.05). The basolateral fraction containing CoQ10 (1.12 μg/mL) and RYRF-derived fraction increased intracellular free cholesterol but remained below the normal-glucose reference (5 mM D-glucose). The basolateral fraction containing cordycepin (0.27 μg/mL) from *Cordyceps sinensis* produced higher intracellular free cholesterol than the normal-glucose control (*p* < 0.001). The combined treatment (containing 0.34 μg/mL cordycepin and 1.58 μg/mL CoQ10) resulted in the highest intracellular free cholesterol levels, significantly exceeding both the untreated high-glucose control and the normal-glucose reference (*p* < 0.001), as well as all individual treatments (*p* < 0.001 vs. RYRF and CoQ10; *p* = 0.009 vs. *Cordyceps sinensis*). Quantitatively, intracellular free cholesterol increased by approximately 1.33-fold relative to the untreated high-glucose control, 32% relative to the normal-glucose control, 47% relative to basolateral fraction containing CoQ10 (1.12 μg/mL) and RYRF-derived fraction, and 15% relative to basolateral fraction containing cordycepin (0.27 μg/mL) from *Cordyceps sinensis* (200 μg/mL). Raw data of intracellular free cholesterol are reported in Appendix B, corresponding to the data presented in Figure 5A.

To further characterise proteins involved in cholesterol handling, ABCA1 protein levels were determined by ELISA (Figure 5D). RYRF-derived fraction produced only a modest increase in ABCA1 protein levels, whereas basolateral fraction containing CoQ10 (1.12 μg/mL) and that containing cordycepin (0.27 μg/mL) from *Cordyceps sinensis* produced significantly higher levels than the untreated high-glucose control (*p* < 0.05). The combined treatment (containing 0.34 μg/mL cordycepin and 1.58 μg/mL CoQ10) resulted in the highest ABCA1 protein levels, corresponding to an increase of approximately 26.5% relative to the untreated high-glucose control and significantly exceeding all individual treatments (*p* < 0.001 vs. RYRF and CoQ10; *p* = 0.005 vs. *Cordyceps sinensis*).

### 2.7. Modulation of the PCSK9–LDLR–SREBP-2 Regulatory Axis

**Figure 6:**
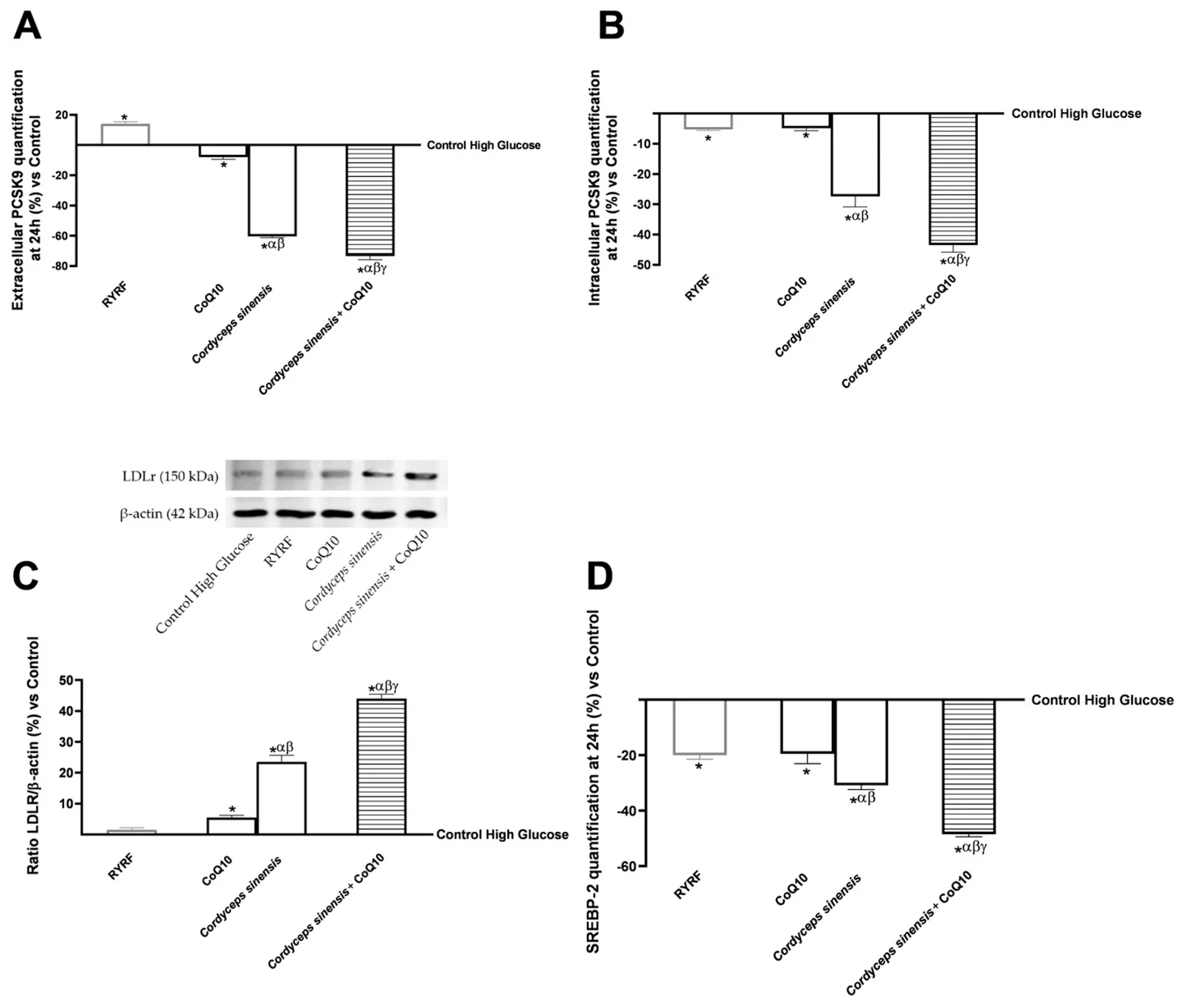
Effects of the tested treatments on the PCSK9–LDLR–SREBP-2 regulatory axis in HepG2 cells maintained under high-glucose conditions following exposure to basolateral supernatants from the intestinal co-culture model. (**A**) Extracellular PCSK9 protein levels determined by ELISA. (**B**) Intracellular PCSK9 protein levels determined by ELISA. (**C**) LDLR protein expression determined by Western blot with representative immunoblot and densitometric analysis. (**D**) SREBP-2 protein levels determined by ELISA. Data are expressed as mean ± SD from five independent biological experiments performed in technical triplicate, except for Western blot analyses, which were performed in three independent biological experiments, each in technical triplicate. Representative Western blot images shown in the figure are from one biological replicate, whereas densitometric data are expressed as mean ± SD from three independent biological experiments. For each biological replicate, β-actin loading controls were obtained from the same experimental membrane as the corresponding target protein analysis. ELISA and densitometric data are presented as percentage change relative to the untreated high-glucose control (0% reference). Statistical significance was determined by one-way ANOVA followed by Dunnett’s post hoc test (ELISA data) or Tukey’s post hoc test (Western blot densitometry). Exact *p*-values are reported in the text. * *p* < 0.05 vs. high-glucose control; α *p* < 0.05 vs. RYRF-derived basolateral fraction (nutraceutical comparator; obtained from 8 μg/mL nominal apical treatment); β *p* < 0.05 vs. CoQ10 basolateral fraction (1.12 μg/mL); γ *p* < 0.05 vs. *Cordyceps sinensis* basolateral fraction (containing 0.27 μg/mL cordycepin).

To further evaluate the coordinated responses associated with LDL handling the PCSK9–LDLR–SREBP-2 regulatory node was evaluated following 24 h exposure of HepG2 cells to basolateral supernatants collected from the intestinal co-culture model (Figure 6).

Extracellular PCSK9 levels were first quantified by ELISA (Figure 6A). RYRF-derived fraction significantly increased extracellular PCSK9 levels compared to the untreated high-glucose control (*p* < 0.05). In contrast, both basolateral fraction containing CoQ10 (1.12 μg/mL) and that containing cordycepin (0.27 μg/mL) from *Cordyceps sinensis* reduced extracellular PCSK9 levels, with *Cordyceps sinensis* producing the greatest reduction among the individual treatments (*p* < 0.001). The combined treatment (containing 0.34 μg/mL cordycepin and 1.58 μg/mL CoQ10) resulted in the lowest extracellular PCSK9 levels, significantly lower than those observed with each individual treatment and the nutraceutical comparator-derived fraction (*p* < 0.001 vs. RYRF and CoQ10; *p* = 0.004 vs. *Cordyceps sinensis*). The combination treatment quantitatively reduced the extracellular PCSK9 levels by approximately 73.5% relative to the untreated high-glucose control, 1.20-fold relative to RYRF-derived fraction, 89% relative to basolateral fraction containing CoQ10 (1.12 μg/mL), and 20% relative to basolateral fraction containing cordycepin (0.27 μg/mL) from *Cordyceps sinensis*. Raw data of intracellular PCSK9 levels are reported in Appendix B, corresponding to the data presented in Figure 6A.

Intracellular PCSK9 protein levels showed a similar pattern (Figure 6B). All treatments significantly reduced intracellular PCSK9 compared to the untreated high-glucose control (*p* < 0.05). Among the individual treatments, basolateral fraction containing cordycepin (0.27 μg/mL) from *Cordyceps sinensis* produced the greatest reduction, whereas the basolateral fraction related to CoQ10 showed values comparable to those of RYRF-derived fraction. The combined treatment (containing 0.34 μg/mL cordycepin and 1.58 μg/mL CoQ10) produced the greatest decrease in intracellular PCSK9, significantly exceeding all individual treatments (*p* < 0.001 vs. RYRF and CoQ10; *p* = 0.005 vs. *Cordyceps sinensis*). The combination treatment quantitatively reduced the intracellular PCSK9 levels by approximately 43% relative to the untreated high-glucose control, 88% relative to both RYRF-derived fraction and basolateral fraction containing CoQ10 (1.12 μg/mL), and 34% relative to basolateral fraction containing cordycepin (0.27 μg/mL) from *Cordyceps sinensis*. Raw data of extracellular PCSK9 levels are reported in Appendix B, corresponding to the data presented in Figure 6A.

LDLR protein levels were subsequently evaluated by Western blot (Figure 6C). All treatments increased LDLR protein levels compared to the untreated high-glucose control (*p* < 0.05). Among the individual treatments, basolateral fraction containing cordycepin (0.27 μg/mL) from *Cordyceps sinensis* produced the greatest increase, whereas basolateral fraction related to CoQ10 showed values comparable to those observed with RYRF-derived fraction. The combined treatment (containing 0.34 μg/mL cordycepin and 1.58 μg/mL CoQ10) resulted in the highest LDLR expression, corresponding to increases of approximately 44% relative to the untreated high-glucose control, 96.5% relative to RYRF-derived fraction, 88% relative to basolateral fraction containing CoQ10 (1.12 μg/mL), and 47% relative to basolateral fraction containing cordycepin (0.27 μg/mL) from *Cordyceps sinensis* (*p* < 0.001 vs. all single agents).

SREBP-2 protein levels were then determined by ELISA (Figure 6D). All individual treatments significantly reduced SREBP-2 protein levels compared to the untreated high-glucose control (*p* < 0.05). The combined treatment (containing 0.34 μg/mL cordycepin and 1.58 μg/mL CoQ10) produced the greatest reduction, corresponding to decreases of approximately 49% relative to the untreated control, 59% relative to RYRF-derived fraction and basolateral fraction containing CoQ10 (1.12 μg/mL), and 37% relative to basolateral fraction containing cordycepin (0.27 μg/mL) from *Cordyceps sinensis* (*p* < 0.001 vs. all single agents).

Taken together, the concurrent reduction in extracellular and intracellular PCSK9, increased total LDLR expression, and enhanced specific DiI-LDL uptake were consistently observed across the assessed endpoints, supporting an overall modulation of LDL handling. To evaluate the internal consistency between our molecular and functional observations, an additional correlation analysis was performed between extracellular PCSK9 levels with total LDLR protein abundance (determined by Western blot) and specific DiI-LDL uptake across the corresponding independent biological replicates. As shown in Supplementary Figure S2, a significant negative Pearson correlation was observed between extracellular PCSK9 levels at 24 h and cellular fluorescent LDL uptake (r = −0.8878, R^2^ = 0.7878, *p* = 0.0002, 95% CI: −0.9550 to −0.7330; Supplementary Figure S2A). Similarly, a remarkably negative correlation was confirmed between extracellular PCSK9 levels and the ratio of LDLR/β-actin protein expression (r = −0.9431, R^2^ = 0.8894, *p* < 0.0001, 95% CI: −0.9776 to −0.8591; Supplementary Figure S2B). This statistical association supports the biological coherence of our findings under the experimental conditions tested, while maintaining a clear distinction between association and direct demonstrated causality.

Finally, extracellular recovery and persistence of intact cordycepin and CoQ10 in the HepG2 culture medium was quantified by RP-HPLC-UV after 24 h of exposure. As summarised in Table A5 (Appendix A), both compounds remained detectable after 24 h of HepG2 exposure in extracellular supernatant. This extracellular persistence corresponded to 14.9% (0.14 μg/mL) for cordycepin and 1.4% (0.62 μg/mL) for CoQ10 (*p* < 0.05). Following administration of the combined treatment, the detectable extracellular concentration in the supernatant increased to 20.2% (0.19 μg/mL) for cordycepin and 2.0% (0.88 μg/mL) for CoQ10, representing increases of approximately 26% and 30%, respectively, compared to the individual treatments (*p* = 0.023). These extracellular measurements indicate the stability and availability of the analytes in the culture environment but do not measure or imply direct intracellular hepatic uptake, intracellular accumulation, or metabolic transformation.

### 2.8. Involvement of AMPK Signalling in the Regulation of Hepatic Cholesterol Metabolism

**Figure 7:**
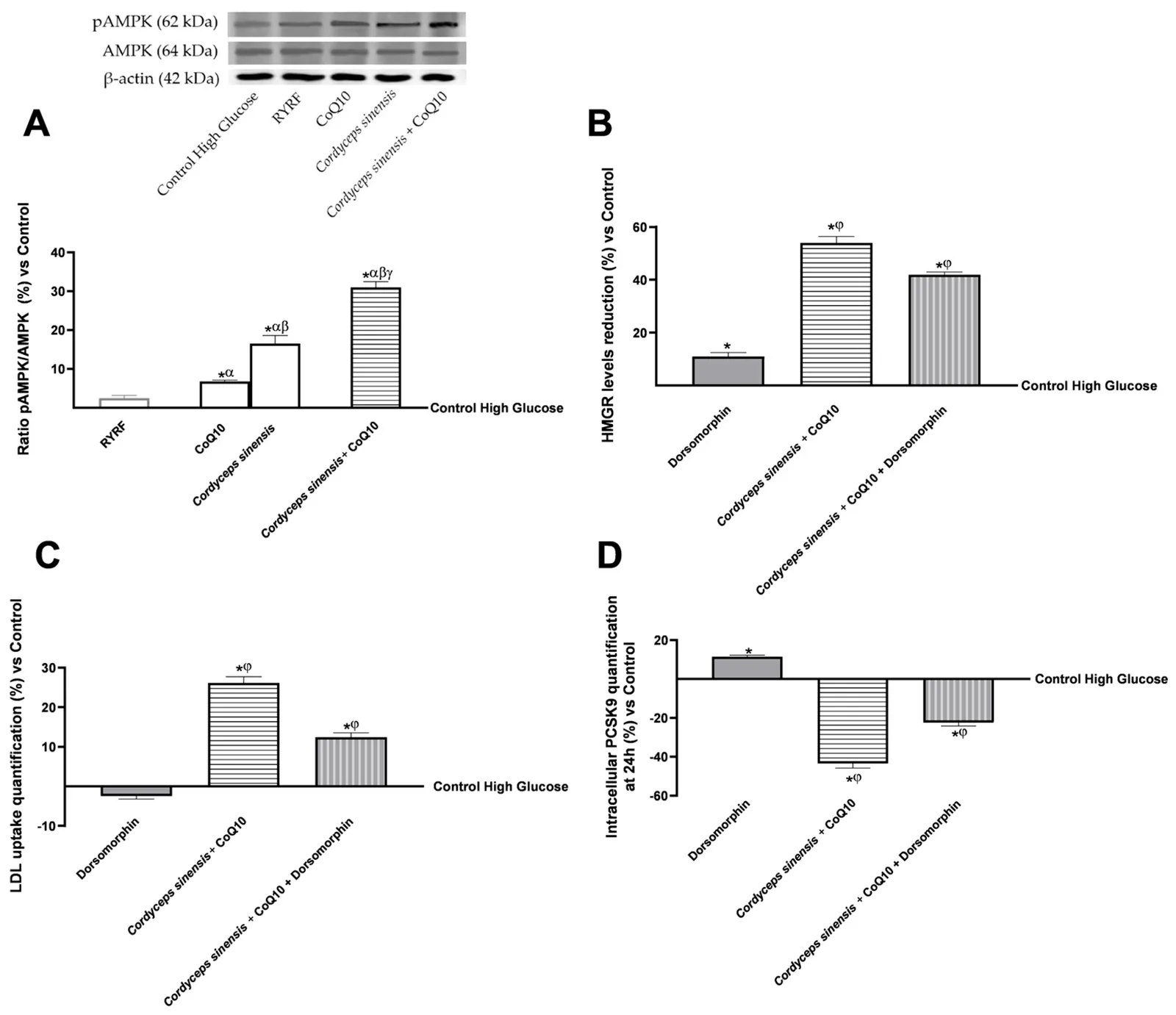
Contribution of AMPK signalling to the hepatic responses induced by *Cordyceps sinensis* and CoQ10 in HepG2 cells maintained under high-glucose conditions. (**A**) AMPK activation determined as the pAMPK/AMPK ratio by Western blot and densitometric analysis. (**B**) HMGR protein levels following treatment in the presence or absence of the AMPK inhibitor dorsomorphin (Compound C). (**C**) PCSK9 protein levels following treatment in the presence or absence of dorsomorphin. (**D**) LDL uptake following treatment in the presence or absence of dorsomorphin. Representative Western blot images shown in the figure are from one biological replicate, whereas densitometric data are expressed as mean ± SD from three independent biological experiments. For each biological replicate, β-actin loading controls were obtained from the same experimental membrane as the corresponding target protein analysis. HMGR, PCSK9, and LDL uptake data are expressed as mean ± SD from five independent biological experiments performed in technical triplicate. All data are presented as percentage change relative to the corresponding untreated high-glucose control. Statistical significance was determined by one-way ANOVA followed by Dunnett’s post hoc test. For panels (**B**–**D**), comparisons between treatments with and without dorsomorphin were evaluated using an unpaired Student’s *t*-test. Exact *p*-values are reported in the text. For panel (**A**): * *p* < 0.05 vs. high-glucose control; α *p* < 0.05 vs. RYRF-derived basolateral fraction (nutraceutical comparator; obtained from 8 μg/mL nominal apical treatment); β *p* < 0.05 vs. CoQ10 basolateral fraction (1.12 μg/mL); γ *p* < 0.05 vs. *Cordyceps sinensis* basolateral fraction (containing 0.27 μg/mL cordycepin). For panels (**B**–**D**): * *p* < 0.05 vs. high-glucose control; φ *p* < 0.05 vs. the corresponding combined basolateral fraction (0.34 μg/mL cordycepin + 1.58 μg/mL CoQ10) without dorsomorphin.

To investigate the potential involvement of AMPK signalling in the hepatic responses induced *by Cordyceps sinensis* and CoQ10, AMPK activation was first evaluated by determining the pAMPK/AMPK ratio in HepG2 cells maintained under high-glucose conditions. These experiments were performed by exposing HepG2 cells to the basolateral intestinal-conditioned medium collected after 6 h of intestinal transport, rather than direct exposure to the original formulations. The contribution of this pathway was subsequently explored using the AMPK inhibitor dorsomorphin (Compound C) (Figure 7).

AMPK activation was assessed by measuring the pAMPK/AMPK ratio (Figure 7A). RYRF-derived fraction produced only a modest increase compared to the untreated high-glucose control. In contrast, basolateral fraction containing CoQ10 (1.12 μg/mL) significantly increased the pAMPK/AMPK ratio relative to both the untreated control and RYRF-derived fraction (*p* < 0.05), whereas basolateral fraction containing cordycepin (0.27 μg/mL) from *Cordyceps sinensis* produced a greater increase than the other individual treatments (*p* < 0.05). The combined treatment (containing 0.34 μg/mL cordycepin and 1.58 μg/mL CoQ10) resulted in the highest pAMPK/AMPK ratio, significantly exceeding all individual treatments and the formulation nutraceutical comparator-derived fraction (*p* < 0.001 vs. RYRF and CoQ10; *p* = 0.001 vs. *Cordyceps sinensis*). Quantitatively, the pAMPK/AMPK ratio increased by approximately 31% relative to the untreated high-glucose control, 90% relative to RYRF-derived fraction, 78% relative to basolateral fraction containing CoQ10 (1.12 μg/mL), and 47% relative to basolateral fraction containing cordycepin (0.27 μg/mL) from *Cordyceps sinensis*.

To further explore the contribution of AMPK signalling, HepG2 cells were pretreated with dorsomorphin (Compound C) before exposure to the combined treatment (containing 0.34 μg/mL cordycepin and 1.58 μg/mL CoQ10). Dorsomorphin pretreatment partially attenuated the reduction in HMGR levels induced by the combined treatment (Figure 7B; *p* = 0.013). Dorsomorphin pretreatment also partially attenuated the reduction in PCSK9 levels induced by the combined treatment (Figure 7C; *p* = 0.003). Dorsomorphin pretreatment significantly attenuated the increase in LDL uptake induced by the combined treatment (Figure 7D; *p* = 0.003). Despite this attenuation, LDL uptake remained significantly higher than that observed in untreated high-glucose control cells.

Overall, dorsomorphin pretreatment partially attenuated the effects of the combined treatment on HMGR, PCSK9, and LDL uptake, supporting a potential contribution of AMPK-related signalling under the present experimental conditions.

To evaluate the internal consistency of our proposed biological framework and explore the statistical association between AMPK activation and the downstream cholesterol-regulating endpoints, Pearson correlation analyses were performed. Individual replicate values of the p-AMPK/AMPK ratio (expressed as percentage versus control) were correlated against the percentage reduction in HMGR levels, extracellular PCSK9 levels, and cellular DiI-LDL uptake. As shown in Supplementary Figure S3, AMPK activation demonstrated a strong and statistically significant correlation with all three parameters. Specifically, a strong positive correlation was observed between AMPK activation and the percentage reduction in HMGR protein levels (r = 0.9552, R^2^ = 0.9125, 95% CI: 0.8881 to 0.9825, *p* < 0.0001; Supplementary Figure S3A). Conversely, a strong negative correlation was found with extracellular PCSK9 levels (r = −0.9370, R^2^ = 0.8780, 95% CI: −0.9752 to −0.8447, *p* < 0.0001; Supplementary Figure S3B). Finally, AMPK activation was almost correlated with the enhancement of cellular DiI-LDL uptake (r = 0.9911, R^2^ = 0.9823, 95% CI: 0.9771 to 0.9966, *p* < 0.0001; Supplementary Figure S3C). These correlations provide robust supportive evidence of a coordinated biological association, although they do not establish direct causality.

## 3. Discussion

Energy and lipid pathways are tightly regulated within the gut–liver axis, where the intestinal epithelium acts as the first barrier for oral compounds, and the liver serves as the main hub for cholesterol metabolism [45,46,47].

In the present study, the combination of *Ophiocordyceps sinensis* and CoQ10 was evaluated using differentiated Caco-2/HT29-MTX co-cultures followed by exposure of HepG2 cells to the corresponding basolateral supernatants. This experimental design allowed the coordinated evaluation of intestinal electrical barrier properties, junctional organisation, paracellular transport, transepithelial recovery of selected bioactive compounds, hepatic redox and signalling responses, and multiple components of cholesterol-related pathways. Within this sequential framework, the incubation times were selected according to the different kinetics of the intestinal and hepatic responses under investigation [48,49,50,51]. The 6 h intestinal transport phase allowed assessment of transepithelial compound transfer while maintaining electrical barrier properties (TEER > 400 Ω·cm^2^), whereas the 24 h hepatic exposure provided a longer timeframe to assess downstream changes in cholesterol-related pathways, including the regulation of key proteins such as HMGR, PCSK9, and LDLR. Although this experimental system does not fully recapitulate the physiological complexity of the gut–liver axis, systemic pharmacokinetics, or inter-organ endocrine regulation, it provides a controlled platform to assess hepatic responses following intestinal passage rather than direct exposure to the original preparations. Preliminary chemical characterisation identified carbohydrates, β-glucan-related fractions, and cordycepin as the principal measurable constituents of the *Ophiocordyceps sinensis* extract. Cordycepin and β-glucans have previously been associated with antioxidant and metabolic effects in experimental systems [52]. These compositional findings provide an analytical framework for interpreting the biological responses, although they do not establish which constituent or combination of constituents was primarily responsible for the observed responses. Moreover, although β-glucans were present in the raw extract, their presence in the basolateral compartment was not directly assessed. Therefore, the hepatic responses cannot be attributed to transepithelial transfer of intact β-glucans and may instead reflect the combined effects of the confirmed compounds cordycepin and CoQ10 and other uncharacterised intestinal-derived constituents.

The use of differentiated Caco-2/HT29-MTX co-cultures on Transwell^®^ inserts provided a physiologically relevant in vitro intestinal model by combining absorptive and mucus-producing epithelial components. This approach permitted a more representative approximation of selected features of the intestinal epithelial environment [48]. This characteristic is particularly relevant to CoQ10, whose high lipophilicity and poor aqueous solubility limit intestinal dissolution and permeability [53]. Previous Caco-2 studies have shown that solubilised, emulsified, or lipid-based delivery systems can increase the apparent permeability and cellular uptake of CoQ10 compared with non-formulated material [54].

Under the experimental conditions, all treatments maintained TEER above the predefined acceptance threshold, while the combined treatment increased TEER and Claudin-1, Occludin, and ZO-1 levels, indicating preserved barrier integrity with modulation of junction-associated proteins [55]. The combination also increased fluorescein transfer across the co-culture. As TEER and tracer flux provide complementary measures of barrier function, this apparent discrepancy may reflect differences in molecular size, transport kinetics, and underlying mechanisms and should not be interpreted as direct evidence of altered tight junction integrity. TEER measures ionic conductance across the paracellular space, primarily reflecting the passage of small inorganic ions such as Na^+^ and Cl^−^, whereas fluorescein permeability assesses the passive paracellular flux of larger, uncharged hydrophilic molecules [56,57]. Thus, the observed increase in fluorescein permeability despite preserved high TEER is mechanistically plausible and may reflect differential regulation of the “pore” and “leak” pathways of tight junctions [58]. While the upregulation of claudin, occludin, and ZO-1 supports the preservation of tight junction organisation and electrical barrier function, selective modulation of the “leak” pathway could facilitate the passage of small hydrophilic molecules such as fluorescein. Potential mechanisms include myosin light chain kinase (MLCK) activation, tricellulin redistribution, or subtle cytoskeletal remodelling; however, these remain plausible hypotheses, as these pathways were not directly assessed in the present study.

RP-HPLC-UV analysis confirmed the recovery of both cordycepin and CoQ10 in the basolateral compartment, with significant recovery following combined administration. Although this does not establish in vivo bioavailability, it demonstrates measurable transepithelial transfer under the experimental conditions. Similar improvements in the transfer of poorly soluble compounds have been reported following modifications of solubilisation and dispersion [59]. This interpretation is further supported by the mass-balance analysis (Appendix A, Table A2), which showed lower CoQ10 recovery when administered alone compared to the combined formulation, consistent with reduced physical losses and improved physicochemical bioaccessibility under the experimental conditions. However, the present data do not allow discrimination among these mechanisms or identification of a specific absorption process.

Intestinal basolateral supernatants were well-tolerated by HepG2 cells and significantly reduced superoxide production under high-glucose conditions, with the combined treatment showing the strongest antioxidant effect. This was accompanied by increased ERK/MAPK activation, particularly following exposure to the *Ophiocordyceps sinensis* and combined fractions. Given the pleiotropic role of ERK/MAPK signalling, this activation may reflect a treatment-associated adaptive response, although its contribution to the observed antioxidant effects remains to be established [49,50,60].

The concomitant preservation of viability and reduction in superoxide production indicate that compounds recovered following intestinal passage retained measurable biological activity in the hepatic compartment. The metabolic and protective effects observed in HepG2 cells were associated with the experimentally recovered basolateral concentrations of cordycepin (0.34 μg/mL) and CoQ10 (1.58 μg/mL) under combined conditions, rather than with the higher nominal concentrations applied apically. These findings suggest that the bioaccessible fractions may retain biological activity after intestinal transport and highlight the relevance of considering experimentally recovered concentrations when interpreting downstream responses in sequential in vitro models. This observation further supports the usefulness of sequential intestinal–hepatic systems for investigating the biological effects of orally relevant compounds after passage through an epithelial barrier [49,61,62]. This pleiotropic modulation is consistent with the multi-target activity described for food-derived bioactives, which can influence interconnected pathways involved in metabolic homeostasis [63]. While our findings are compatible with this concept, mechanisms reported for other bioactives cannot be directly extrapolated to the *Ophiocordyceps sinensis*–CoQ10 formulation. Rather, they provide a biological context for the coordinated pathway modulation observed here. Moreover, as intestinal processing and bioaccessibility can influence the activity of orally administered compounds [64] the sequential model used here provides a relevant framework for assessing their downstream hepatic effects. It does not, however, establish their metabolic fate, systemic bioavailability, or in vivo activity following oral administration. Although transepithelial recovery of cordycepin and CoQ10 was confirmed by HPLC, the hepatic effects reflect the complete basolateral intestinal-conditioned medium and cannot be attributed solely to these compounds. RP-HPLC-UV analysis (Table A4) confirmed their extracellular persistence after 24 h; however, changes in medium levels cannot be interpreted as direct evidence of hepatic uptake.

High-glucose exposure is commonly used to induce metabolic and oxidative stress in hepatic cell models and can alter glucose and lipid-related pathways [65,66]. In the present study, this condition was used to establish a metabolically challenged environment rather than to reproduce hypercholesterolaemia itself. To distinguish glucotoxicity from non-specific osmotic stress, physiological glucose (5 mM) and hyperosmotic (5 mM glucose + 24.5 mM mannitol) controls were included. The absence of significant metabolic changes in the mannitol group (Supplementary Table S1) supports a glucose-specific modulation of the observed responses. Within this context, the combination of *Ophiocordyceps sinensis* and CoQ10 affected several interconnected components of hepatic cholesterol-related pathways more strongly than either individual treatment or RYRF nutraceutical comparator.

Reduced HMGR, SREBP-2, and total intracellular cholesterol levels were consistent with the attenuation of cholesterol biosynthesis, although enzymatic activity and SREBP-2 processing were not directly assessed. These findings therefore indicate modulation of proteins associated with cholesterol synthesis rather than direct changes in biosynthetic flux. The combined treatment also reduced intracellular and extracellular PCSK9 levels, increased LDLR abundance, and enhanced DiI-LDL uptake, supporting increased receptor-mediated LDL internalisation. Together, the opposing changes in PCSK9 and LDLR indicate a coherent modulation of LDL receptor availability. This interpretation is further supported by the significant negative correlations between extracellular PCSK9 levels and both DiI-LDL uptake (r = −0.8878) and total LDLR abundance (r = −0.9431) (Supplementary Figure S2). However, the canonical PCSK9–LDLR pathway involves multiple steps that were not directly assessed here, including receptor internalisation, recycling, turnover, and PCSK9-dependent degradation; the observed changes should be interpreted as a coordinated biological association rather than a demonstrated causal sequence [67]. LDL-associated cholesterol was not considered a direct measure of intact LDL particles or receptor trafficking and was therefore validated by the receptor-specific DiI-LDL uptake assay. Furthermore, since cell-surface LDLR abundance, receptor trafficking, turnover, and direct PCSK9-dependent degradation were not assessed, the observed association between reduced PCSK9, increased LDLR availability, and enhanced DiI-LDL uptake cannot be interpreted as a direct causal sequence. Receptor-independent uptake pathways cannot be excluded.

RYRF showed a distinct response profile, characterised by increased extracellular PCSK9 and more modest effects on LDLR and LDL uptake. Given that statins can induce PCSK9 through SREBP-2-dependent mechanisms [65], this profile shares some features of a statin-associated response. However, RYRF was not chemically or pharmacologically equivalent to a purified statin and should not be considered representative of clinical statin treatment.

Additional analyses suggested modulation of pathways involved in cholesterol catabolism and cellular cholesterol handling. Increased intracellular total bile acids and CYP7A1 protein levels were consistent with modulation of the pathway converting cholesterol to bile acids; however, these static measures cannot establish increased bile acid synthesis without direct assessment of enzymatic activity or metabolic flux [68]. Similarly, increased ABCA1 protein levels, particularly following the combined treatment, suggested modulation of cholesterol efflux-related pathways [68]. Since transporter or receptor abundance does not necessarily reflect functional activity or trafficking, these findings cannot be considered direct evidence of increased cholesterol export or LDLR-mediated uptake without functional assays.

Since cholesterol esterification and intracellular distribution were not directly assessed, the observed accumulation, together with increased CYP7A1 and ABCA1 levels, may reflect a redistribution of cholesterol toward metabolically active pools rather than a net increase in intracellular sterol content. Notably, the more pronounced hepatic responses observed with the combined treatment were consistent with the exploratory Bliss independence analyses of cholesterol and bile acid levels. This finding supports greater-than-additive mathematical interaction under the specific experimental conditions but does not, by itself, establish definitive pharmacological synergy or define the molecular basis of the interaction. Furthermore, as the Bliss analysis was based on a single concentration combination without formal statistical comparison between observed and predicted values, these findings should be considered exploratory. Comprehensive concentration–response studies, including isobolographic or checkerboard analyses, will be required to characterise the pharmacological interaction of the combination.

AMPK signalling represents a plausible link between cellular energy status and cholesterol metabolism, as its activation can suppress anabolic lipid pathways and promote metabolic adaptation [69,70]. The combined treatment induced the greatest increase in the pAMPK/AMPK ratio, together with reduced HMGR and PCSK9 levels and increased LDL uptake. Dorsomorphin partially attenuated these effects, supporting a possible involvement of AMPK signalling. Correlation analyses (Supplementary Figure S3) further supported associations between AMPK activation and changes in HMGR, PCSK9, and LDL uptake. However, dorsomorphin is not a selective AMPK inhibitor and has documented off-target effects [71]; therefore, these findings should be considered supportive rather than definitive evidence of AMPK involvement. Confirmation of a causal role will require genetic approaches or more selective AMPK modulators.

The persistence of measurable effects in the presence of dorsomorphin suggests that the observed response is not exclusively dependent on AMPK. This is consistent with the pleiotropic activities reported for cordycepin, β-glucans, and CoQ10, which can modulate interconnected redox, mitochondrial, inflammatory, and lipid-related pathways [72,73,74]. The ERK/MAPK response may represent an additional component of this broader signalling profile, although its specific role in cholesterol regulation remains unclear.

The combined treatment may reflect complementary activities of its constituents, providing biological plausibility for the observed effects on oxidative stress and energy- and lipid-related signalling [72,73,74]. However, as the experiments used the complete extract in combination with CoQ10, the individual contributions of cordycepin, β-glucans, other extract components, and their potential interactions cannot be distinguished.

To the best of our knowledge, this is the first study to investigate the combination of *Ophiocordyceps sinensis* and CoQ10 using sequential exposure of a differentiated Caco-2/HT29-MTX intestinal model and HepG2 cells, integrating compound recovery with the assessment of hepatic cholesterol-related pathways. HPLC-based detection of cordycepin and CoQ10 after intestinal passage and hepatic exposure strengthens the link between the analytical and biological components of the model. These findings provide a preclinical rationale for further investigation, but do not establish efficacy, intestinal absorption, or cholesterol-lowering effects in humans.

Several limitations should be considered. The use of immortalised cell lines does not reproduce the cellular heterogeneity, three-dimensional architecture, or inter-organ interactions of the in vivo environment. Although the Caco-2/HT29-MTX model incorporates a mucus-producing component, it lacks immune, vascular, stromal, neuronal, and microbiota-derived influences, while HepG2 cells differ from primary hepatocytes in key aspects of hepatic metabolism and lipoprotein handling. The current model does not fully account for the highly lipophilic nature of CoQ10 and the crucial rate-limiting role of micellization in its intestinal absorption, which is a key constraint previously highlighted in the literature [53,54]. In this context, the lack of direct physicochemical characterisation of CoQ10 under cell culture conditions remains a limitation, despite increased CoQ10 detection with *Ophiocordyceps sinensis* (Table A2). Such analyses are necessary to distinguish formulation effects from direct cellular modulation and represent important directions for future studies [53,54].

Moreover, the sequential model captures selected features of intestinal passage and hepatic exposure but does not account for luminal digestion, systemic distribution, first-pass metabolism, protein binding, elimination, or whole-body pharmacokinetics. Therefore, the in vitro concentrations cannot be directly extrapolated to hepatic exposure following oral administration in humans. The sequential design did not evaluate independent dose–response profiles of the nominal formulations in HepG2 cells. Although this is a limitation, direct exposure to raw compounds would bypass intestinal transport and thus would not reflect the primary objective of assessing hepatic responses following epithelial processing.

Third, several endpoints relied on protein abundance or static biochemical measurements rather than direct assessment of metabolic flux. HMGR and CYP7A1 activities, LDLR surface localisation and turnover, cholesterol esterification and efflux, and individual bile acid species were not directly measured. In addition, the high-glucose model represents a simplified metabolic stress condition and does not recapitulate the multifactorial pathophysiology of dyslipidaemia, metabolic dysfunction-associated steatotic liver disease (MASLD), or hypercholesterolaemia. Comparison with RYRF, which is a clinically relevant nutraceutical comparator [9,12], should be interpreted cautiously, as its concentration was based on non-cytotoxic MTT thresholds rather than normalised monacolin K content. Thus, the observed differences reflect distinct in vitro biological responses and do not indicate greater cholesterol-lowering potency or clinical efficacy.

Finally, dorsomorphin alone cannot establish AMPK-specific causality, and the individual contributions of *Ophiocordyceps sinensis* constituents to the combined response remain unresolved. These limitations should guide the design of subsequent studies incorporating primary cells or advanced three-dimensional models, genetic interrogation of AMPK signalling, direct measurements of cholesterol flux, and in vivo evaluation of pharmacokinetics, safety, and lipid-related outcomes. These observations support further preclinical investigation of the combination, while emphasising the need for direct metabolic flux studies and in vivo validation before conclusions can be drawn regarding its physiological or clinical relevance. Since the composition of the transferred basolateral intestinal-conditioned medium remains partly uncharacterised, this represents a further limitation of the present study. Future targeted and untargeted chemical, metabolomic, and proteomic analyses could help define its composition and clarify the contribution of individual components to the observed hepatic responses.

Overall, the present findings indicate that compounds recovered after intestinal exposure to *Ophiocordyceps sinensis* and CoQ10 retained measurable biological activity following epithelial passage and modulated multiple interconnected pathways involved in hepatic cholesterol-related pathways under high-glucose conditions. The combined treatment was associated with reductions in HMGR, total cholesterol, PCSK9, and SREBP-2, and increases in LDLR, LDL uptake, CYP7A1, intracellular bile acid levels, ABCA1, and AMPK activation. These observations support further preclinical investigation of the combination, while emphasising the need for direct metabolic flux studies and in vivo validation before conclusions can be drawn regarding its physiological or clinical relevance.

## 4. Materials and Methods

### 4.1. Chemical Characterisation of Ophiocordyceps sinensis Extract

Chemical characterisation of the *Ophiocordyceps sinensis* extract was performed using complementary analytical methods to quantify selected carbohydrate fractions, β-glucans, and cordycepin. This independent analytical characterisation was performed in our laboratory prior to the biological experiments to establish an experimental quality-control procedure, thereby distinguishing our laboratory-verified data from the baseline batch specifications provided by the manufacturer. All analyses were conducted on the same batch of extract used in the biological experiments.

#### 4.1.1. Determination of Total Carbohydrate Content

Total carbohydrate content was determined using the phenol–sulfuric acid method, as previously described [75,76]. Briefly, 6 mg of *Ophiocordyceps sinensis* extract was dissolved in 1 mL of deionised water and mixed with 1 mL of a 5% (*w*/*w*) aqueous phenol solution prepared from redistilled phenol (Merck Life Science, Rome, Italy). Concentrated sulfuric acid (5 mL; 99%; Merck Life Science, Rome, Italy) was then rapidly added to the mixture. After incubation for 10 min at room temperature, the samples were vortexed for 30 s and incubated for a further 20 min to allow colour development. Absorbance was measured at 490 nm using an Infinite 200 PRO MPlex microplate reader (Tecan, Männedorf, Switzerland). Quantification was performed using a calibration curve generated from sucrose standard solutions over the concentration range of 10–70 mg/L. Analyses were conducted in triplicate, and results are expressed as mean ± SD (% *w*/*w*).

#### 4.1.2. Determination of β-1,3/1,6-Glucans and Total β-1,3-Glucans

β-1,3/1,6-glucans were estimated using a colourimetric method based on the interaction of Congo red with β-glucans in their triple-helical conformation [77]. Measurements were performed at pH 7. An aliquot of 100 μL of sample or standard solution was diluted with distilled water to a final volume of 700 μL. Subsequently, 600 μL of 0.2 mol/L citrate/sodium hydroxide buffer at pH 7 (Merck Life Science, Rome, Italy) and 100 μL of Congo red solution were added. The Congo red solution was prepared by dissolving 0.08 g of analytical-grade Congo red (Merck Life Science, Rome, Italy) in 100 mL of buffer. After thorough mixing, 100 μL aliquots were transferred in triplicate to a 96-well plate, and absorbance was measured at 523 nm using an Infinite 200 PRO MPlex microplate reader (Tecan, Männedorf, Switzerland). Quantification was performed using a calibration curve generated with schizophyllan (Carl ROTH, Karlsruhe, Germany), a branched β-1,3/1,6-glucan derived from *Schizophyllum commune*, over the concentration range of 0–150 μg/mL. Results are expressed as mean ± SD (% *w*/*w*).

Total β-1,3-glucan content was determined using a fluorimetric method based on aniline blue staining, as previously described [78]. A dye mixture containing 0.1% (*w*/*v*) aniline blue in water, 1 N HCl, and 1 M glycine/NaOH buffer at pH 9.5 (Merck Life Science, Rome, Italy), combined at a volume ratio of 40:21:59, was added to the *Ophiocordyceps sinensis* extract. Samples were incubated at 50 °C for 30 min to allow formation of the fluorescent complex between β-1,3-glucans and aniline blue, then maintained at room temperature for a further 30 min before measurement.

Curdlan (Merck Life Science, Rome, Italy) was used as the reference standard to generate a calibration curve over the concentration range of 1–30 μg/mL. Fluorescence was measured using an Infinite 200 PRO MPlex microplate reader (Tecan, Männedorf, Switzerland) at excitation and emission wavelengths of 392 and 502 nm, respectively. Analyses were conducted in triplicate, and results are expressed as mean ± SD (% *w*/*w*).

#### 4.1.3. HPLC Determination of Cordycepin in the Fungal Extract

Cordycepin was quantified using RP-HPLC-UV (Agilent Technologies, Santa Clara, CA, USA). Chromatographic separation was performed using a reversed-phase C18 column (150 × 4.6 mm; particle size, 5 μm) maintained at 30 °C, and detection was carried out at 260 nm. The mobile phase consisted of water as solvent A and methanol as solvent B, delivered at a flow rate of 1.0 mL/min. The gradient elution programme began at 10% solvent B, increased linearly to 50% B over 10 min, was maintained at 50% B for 5 min, and then returned to the initial conditions for column re-equilibration. The total run time was 20 min. A cordycepin stock standard solution (Merck Life Science, Rome, Italy) was prepared at 1 mg/mL by dissolving an accurately weighed amount of cordycepin in methanol (Merck Life Science, Rome, Italy). Samples were prepared by accurately weighing 100 mg of dried *Ophiocordyceps sinensis* extract, extracting it with 10 mL of methanol by sonication for 30 min, and filtering the resulting solution through a 0.45 μm membrane filter before injection. The calibration curve was generated by plotting chromatographic peak area against cordycepin concentration over the range of 5–100 μg/mL and showed a coefficient of determination of R^2^ > 0.999. Cordycepin was identified by comparison with the analytical standard and showed a retention time of 8.8–9.0 min. Analyses were performed in triplicate, and results are expressed as mean ± SD (% *w*/*w*). The chromatographic conditions described above were used exclusively for the characterisation and standardisation of the raw *Ophiocordyceps sinensis* extract. For the determination of cordycepin in biological samples (basolateral transport and post-hepatic supernatants), a dedicated, validated low-range RP-HPLC-UV method preceded by solid-phase extraction (SPE) was employed, as described in Section 4.1.4.

#### 4.1.4. Low-Range HPLC Determination of Cordycepin in Biological Samples

For the quantification of cordycepin in transport and hepatic samples, a low-range RP-HPLC-UV (Agilent Technologies, Santa Clara, CA, USA) method was developed and validated. Basolateral and hepatic supernatants (500 μL) were subjected to C18 solid-phase extraction (SPE; 100 mg, 1 mL; Macherey-Nagel, Düren, Germany). Cartridges were conditioned with 1 mL methanol and 1 mL deionized water, after which samples were loaded, washed with 1 mL water, and eluted with 1 mL of 50% (*v*/*v*) methanol (Merck Life Science, Rome, Italy) in water. The eluates were evaporated under nitrogen at 40 °C and reconstituted in 100 μL mobile phases, corresponding to a five-fold concentration. Samples (20 μL) were analysed using the chromatographic system and column described in Section 4.1.3, with isocratic water:methanol (90:10, *v*/*v*) at 1.0 mL/min and UV detection at 260 nm. Quantification was based on an external calibration curve prepared in blank culture medium and processed using the same SPE procedure over 0.05–2.00 μg/mL (R^2^ = 0.9994). Validation parameters (LOD, LOQ, recovery, and precision) are reported in Supplementary Table S3. All analytical determinations were performed in triplicate, and results are expressed as mean ± SD (μg/mL).

### 4.2. HPLC Determination of CoQ10

CoQ10 was quantified using high-performance liquid chromatography coupled with ultraviolet–visible (HPLC-UV; Agilent Technologies, Santa Clara, CA, USA) detection, as previously described [79]. Chromatographic separation was performed using a reversed-phase C18 column (250 × 4.6 mm; particle size, 5 μm) maintained at ambient temperature. The mobile phase consisted of methanol: isopropanol (70:30, *v*/*v*; Merck Life Science, Rome, Italy) and was delivered isocratically at a flow rate of 1.0 mL/min. Samples were centrifuged at 12,000 rpm for 10 min and filtered through a 0.22 μm polytetrafluoroethylene syringe filter (Merck Life Science, Rome, Italy) before injection. The injection volume was 20 μL, and detection was performed at 275 nm. CoQ10 was identified by comparing its retention time with that of an external analytical standard. Quantification was based on an external calibration curve generated over the concentration range of 0.1–50 μg/mL, which showed a coefficient of determination of R^2^ > 0.999. Analytical performance was evaluated in terms of linearity, intra-day and inter-day precision, limit of detection (LOD), limit of quantification (LOQ), and recovery from biological matrices. Intra-day and inter-day precision showed relative standard deviations below 10%, whereas recovery from biological matrices exceeded 85%. The LOD and LOQ were 0.1 and 0.3 μg/mL, respectively. Results are expressed as μg/mL, and all analytical determinations were performed in triplicate. To assess CoQ10 recovery within the sequential gut–liver model, CoQ10 concentrations were measured in the basolateral compartment of the intestinal model and in the culture medium collected following HepG2 exposure.

### 4.3. Preparation of Test Compounds

The test materials consisted of *Ophiocordyceps sinensis* extract and CoQ10 (yellow/orange crystalline powder with particle size of 80 mesh, purity 99.4%, and melting point of 51–52 °C). The fungal material studied was a dry extract obtained from the cultured mycelium of *Paecilomyces hepiali* (fungal strain isolated from natural *Ophiocordyceps sinensis*) through a standardised process of hot water extraction, followed by filtration, concentration and spray drying. According to the manufacturer’s specifications, the extract batch was standardised to contain a β-glucan content of ≥40%. To ensure experimental reproducibility and batch consistency, the specific batch used in this study complied with the manufacturer’s quality control and purity standards. The batch met predefined criteria for particle size, moisture content, total ash, tapping density, heavy metal limits, microbiological quality (including total aerobic microbial count, yeast/mould count, and no pathogens), and pesticide residue limits.

An RYRF-based reference formulation, consisting of an *Oryza sativa* L. extract standardised to 5.2% total monacolins, was included as the nutraceutical comparator. All test materials were supplied by noiVita S.r.l.s. (Novara, Italy).

The *Ophiocordyceps sinensis* extract (which was further characterised in our laboratory prior to biological evaluation, as described in Section 4.1) was dispersed directly in phenol red-free Dulbecco’s Modified Eagle’s Medium (DMEM; Merck Life Science, Milan, Italy) supplemented with 2 mM L-glutamine and 1% penicillin–streptomycin. CoQ10 stock solutions were prepared in ethanol (Merck Life Science, Rome, Italy) and subsequently diluted in the appropriate culture medium to obtain the required final concentrations. The final ethanol concentration in the culture medium did not exceed 0.001% (*v*/*v*). Matched vehicle controls containing the corresponding ethanol concentration were included in all experiments involving CoQ10. The physical state of CoQ10 after dilution in aqueous apical medium was not directly characterised. Potential losses due to precipitation or adsorption were therefore assessed through the mass-balance analysis described in Section 4.7.2.

The concentration ranges were selected based on preliminary cytocompatibility experiments performed in the intestinal model and on concentrations previously reported in relevant in vitro studies [66,80,81]. The concentrations evaluated were 100, 200, and 300 μg/mL for *Ophiocordyceps sinensis* extract; 4.3, 13, and 43 μg/mL for CoQ10; and 8, 80, and 160 μg/mL for RYRF. Based on the preliminary cytocompatibility and intestinal barrier experiments, the concentrations selected for subsequent intestinal and sequential gut–liver experiments were 200 μg/mL for *Ophiocordyceps sinensis* extract, 43 μg/mL for CoQ10, and 8 μg/mL for RYRF. For the combined treatment, *Ophiocordyceps sinensis* extract and CoQ10 were simultaneously applied at 200 and 43 μg/mL, respectively.

### 4.4. Cell Culture Model

#### 4.4.1. Intestinal Cell Model

The intestinal model consisted of a co-culture of human Caco-2 enterocyte-like cells and HT29-MTX mucus-secreting goblet-like cells, both obtained from the American Type Culture Collection (ATCC, Manassas, VA, USA). Cells were maintained in Advanced DMEM/F-12 medium (Gibco, Thermo Fisher Scientific, Waltham, MA, USA) supplemented with 10% foetal bovine serum, 2 mM L-glutamine, and 1% penicillin–streptomycin at 37 °C in a humidified atmosphere containing 5% CO_2_. Caco-2 cells were used between passages 26 and 32, whereas HT29-MTX cells were used between passages 10 and 20 to minimise passage-related variability and preserve their mucus-secreting phenotype [82]. For intestinal barrier and transport experiments, Caco-2 and HT29-MTX cells were seeded at a 9:1 ratio onto 6.5 mm Transwell^®^ inserts fitted with 0.4 μm pore-size polycarbonate membranes (Corning Costar, New York, NY, USA). Co-cultures were maintained for 21 days to promote epithelial differentiation and mucus layer formation [48]. Model maturation and electrical barrier properties were monitored by TEER measurements.

For preliminary cytocompatibility experiments, Caco-2/HT29-MTX co-cultures were seeded in 96-well plates at a density of 1 × 10^4^ cells/well and evaluated using the MTT assay.

#### 4.4.2. Hepatic Cell Model

The human hepatoma-derived HepG2 cell line was obtained from ATCC and maintained in DMEM supplemented with 10% foetal bovine serum, 2 mM L-glutamine, and 1% penicillin–streptomycin at 37 °C in a humidified atmosphere containing 5% CO_2_ [83]. Experiments were initiated when cells reached 90–95% confluence to reduce variability associated with cell density [84].

For the sequential gut–liver experiments, HepG2 cells were seeded onto 6.5 mm Transwell^®^ permeable supports fitted with 0.4 μm pore-size membranes (Corning Costar, New York, NY, USA). This compartmentalised configuration enabled controlled exposure of HepG2 cells to basolateral fractions recovered from the intestinal model and reproduced selected aspects of sequential intestinal-to-hepatic exposure [85]. No additional differentiation protocol was applied to HepG2 cells [86]. To establish a metabolically stressed high-glucose condition, HepG2 cells were maintained in medium containing 30 mM D-glucose (Merck Life Science, Milan, Italy) throughout the experiments. For physiological glucose control experiments, cells were maintained in medium containing 5 mM D-glucose. To control for potential hyperosmolar effects, an additional osmotic control group was established by maintaining HepG2 cells in medium containing 5 mM D-glucose and 24.5 mM D-mannitol (Merck Life Science, Milan, Italy) and reported in Supplementary Materials.

Control HepG2 cells were maintained under the same high-glucose conditions and exposed to basolateral fractions collected from untreated intestinal co-cultures [83].

### 4.5. Experimental Design and Sequential Gut–Liver Model

The experimental workflow comprised three consecutive phases: chemical characterisation of the *Ophiocordyceps sinensis* extract, assessment of intestinal cytocompatibility, electrical barrier properties, paracellular transport, and transepithelial recovery, and evaluation of hepatic oxidative status and cholesterol-related pathways following exposure to intestinal basolateral fractions. During the first phase, the *Ophiocordyceps sinensis* extract was chemically characterised for total carbohydrate content, β-glucans, and cordycepin before biological testing.

During the intestinal phase, differentiated Caco-2/HT29-MTX co-cultures were exposed apically to *Ophiocordyceps sinensis* extract, CoQ10, their combination, or RYRF for 1 h to 6 h. The selection of compound concentrations was intentionally performed during a dose–response phase, where concentration-dependent effects (100–300 μg/mL for *Ophiocordyceps sinensis*; 4.3–43 μg/mL for CoQ10; 8–160 μg/mL for RYRF) on epithelial cytocompatibility was first evaluated to ensure that basolateral fractions were generated under non-toxic conditions that preserved junctional sealing. After that, the selected concentrations were used in subsequent experiments comparing the individual treatments with the combination in the same cellular model. Cytocompatibility, TEER, tight junction protein levels, fluorescein transfer, and basolateral recovery of cordycepin and CoQ10 were evaluated. The 6 h intestinal transport phase was selected based on preliminary kinetic optimisation experiments and published studies using differentiated Caco-2/HT29-MTX models [48,51]. This duration provided measurable transepithelial transfer while preserving cellular metabolic activity (MTT assay; Merck Life Science, Milan, Italy) and electrical barrier properties (TEER > 400 Ω·cm^2^), and limiting potential effects associated with prolonged apical exposure.

After 6 h of intestinal exposure, 500 μL of each basolateral intestinal-conditioned medium (containing analytically confirmed transported compounds and other uncharacterised intestinal-derived constituents) was immediately transferred, without dilution, storage, or freezing, to the HepG2 cultures, which were then exposed for 24 h before endpoint assessment. This transport-transfer protocol was designed to expose the hepatic compartment exclusively to the bioaccessible fractions generated following intestinal epithelial processing, rather than to the nominal concentrations initially applied to the apical compartment, thereby maintaining the sequential design of the in vitro model. The 24 h hepatic incubation period was selected to allow sufficient time for the assessment of transcriptional and protein-level responses related to cholesterol-related pathways, while limiting potential secondary effects and the loss of cell viability associated with prolonged exposure to high-glucose conditions [49,50,72].

During the hepatic phase, HepG2 cells maintained under high-glucose conditions were exposed to basolateral fractions collected from the intestinal co-culture following epithelial processing and transepithelial passage of the test compounds. The principal endpoints included cellular metabolic activity (MTT assay), superoxide production, intracellular and extracellular LDL-associated cholesterol, LDL uptake, total and free intracellular cholesterol, and intracellular bile acid levels. Proteins associated with hepatic cholesterol synthesis, uptake, efflux, and conversion into bile acids were also evaluated, including HMGR, LDLR, PCSK9, SREBP-2, ABCA1, and CYP7A1.

AMPK signalling was investigated as exploratory mechanistic endpoints. AMPK was selected because of its established role in cellular energy sensing and previous evidence of its modulation by cordycepin and other *Cordyceps*-derived compounds. Dorsomorphin (Merck Life Science, Milan, Italy) was subsequently used as an exploratory pharmacological approach to assess whether selected cholesterol-related responses were sensitive to the inhibition of AMPK-associated signalling.

To ensure biological reproducibility, major cellular experiments were performed using five independent biological replicates (*n* = 5), each conducted with independently prepared, passage-matched cell cultures. Western blot analyses included three independent biological experiments (*n* = 3). Treatment-associated responses showed consistent directionality and comparable effect sizes across independent experiments, supporting the reproducibility of the experimental findings.

**Figure 8:**
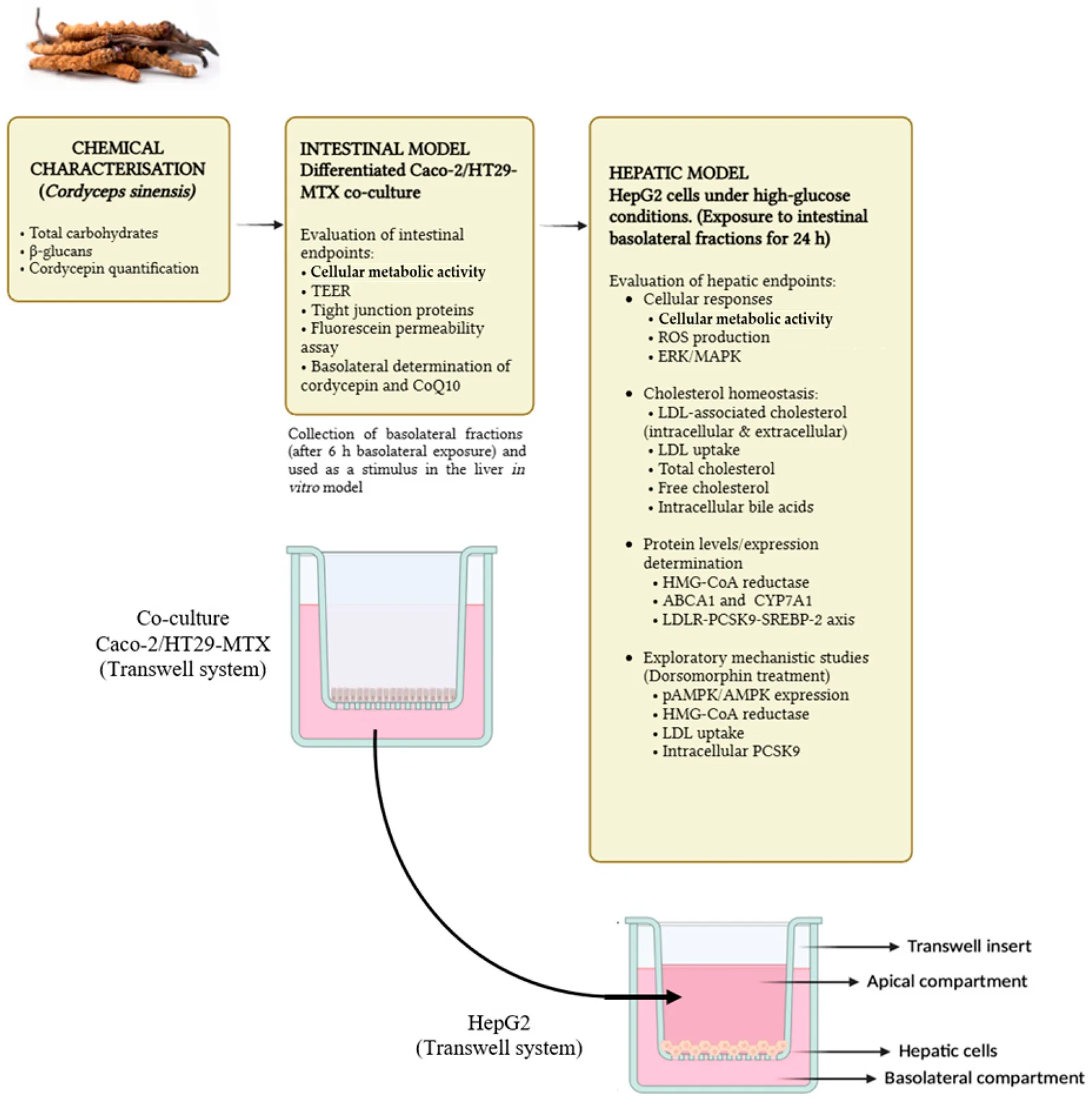
Experimental workflow of the sequential gut–liver model.

A schematic overview of the experimental workflow is provided in Figure 8.

### 4.6. Shared Cellular Assays

#### Cellular Metabolic Activity

Cellular metabolic activity was evaluated in both the intestinal and hepatic models using the MTT In Vitro Toxicology Assay Kit (Merck Life Science, Milan, Italy), according to a previously described protocol [87].

Following treatment, the resulting formazan crystals were solubilised, and absorbance was measured at 570 nm with background correction at 690 nm using an Infinite 200 PRO MPlex microplate reader (Tecan, Männedorf, Switzerland). Technical replicates were averaged within each independent experiment before statistical analysis.

To exclude potential abiotic interference with the MTT assay, cell-free controls were performed by incubating all sample directly with MTT under the same experimental conditions. Tetrazolium reduction was negligible in all treatments (ΔOD570 < 0.01), corresponding to <1% of the typical cellular signal and confirming the absence of significant assay interference.

Data was normalised to the corresponding untreated control and expressed as a percentage of control. Data are reported as mean ± SD from five independent biological experiments, each performed in technical triplicate.

### 4.7. Intestinal Barrier and Transepithelial Recovery

#### 4.7.1. In Vitro Intestinal Barrier Model

The intestinal barrier model was established using differentiated Caco-2/HT29-MTX co-cultures grown on Transwell^®^ inserts to evaluate electrical barrier properties, paracellular transport, and compound recovery [82]. Caco-2 and HT29-MTX cells were seeded at a 9:1 ratio onto 6.5 mm Transwell^®^ inserts fitted with 0.4 μm pore-size polycarbonate membranes (Corning Costar, New York, NY, USA) and maintained for 21 days with regular medium replacement in both the apical and basolateral compartments to promote epithelial differentiation and mucus layer formation, according to established protocols [51,80].

TEER measurements were performed before treatment and at each experimental time point to monitor electrical barrier properties. Only inserts exhibiting baseline TEER values of at least 400 Ω·cm^2^ were included in the study. TEER values were corrected by subtracting the resistance of cell-free inserts and multiplying the resulting value by the membrane surface area. Results were expressed as Ω·cm^2^ and as percentage change relative to the corresponding untreated control. Before treatment, the apical medium was set to pH 6.5, while the basolateral medium was kept at pH 7.4. The co-cultures were equilibrated for 15 min at 37 °C in a humidified atmosphere containing 5% CO_2_ before apical exposure to the test compounds for 1–6 h. Paracellular permeability was evaluated using fluorescein (0.04%; Santa Cruz Biotechnology, Santa Cruz, CA, USA) as a low-molecular-weight permeability marker [88]. At each experimental time point, fluorescein was added to the apical compartment, and the co-cultures were incubated for 40 min at 37 °C. Fluorescence in the basolateral compartment was subsequently measured using an Infinite 200 PRO MPlex microplate reader (Tecan, Männedorf, Switzerland) at excitation and emission wavelengths of 490 and 514 nm, respectively. Basolateral fluorescence was used as an index of transepithelial permeability.

To obtain an absolute quantitative value, the *Papp* values expressed in cm/s was calculated using the following standard equation [89]:

$$P_{app}=\frac{dQ}{dt}\times \frac{1}{A\times C_{0}}$$

where *dQ/dt* is the transport rate of the fluorescent tracer across the monolayer (calculated as the amount of fluorescein recovered in the basolateral compartment over the incubation time), *A* is the surface area of the Transwell insert filter (0.33 cm^2^), and C_0_ is the initial concentration of fluorescein added to the apical chamber (0.04%).

Cell-free Transwell^®^ inserts were included as membrane controls. Results are expressed as percentage change relative to the untreated control and reported as mean ± SD from five independent biological experiments, each performed in technical triplicate.

#### 4.7.2. Mass-Balance Analysis

At the end of the 6 h transport incubation, a complete mass-balance analysis of cordycepin and CoQ10 was performed. The apical medium was collected, and the cells were washed with ice-cold phosphate-buffered saline (PBS; Merck Life Science, Milan, Italy). To recover the cell-associated and membrane-bound fractions, the polycarbonate membrane filter was excised from the Transwell insert and lysed in methanol under sonication for 15 min. Cordycepin and CoQ10 were quantified in the apical, cell-associated/membrane-bound, and basolateral fractions using the HPLC-UV methods described in Section 4.1.3 and Section 4.2. The total mass balance was calculated using the following equation:

$$\mathrm{Mass}\;\mathrm{Balance}\;(\%)=\frac{M_{\mathrm{apical}}+M_{\mathrm{cell}/\mathrm{membrane}}+M_{\mathrm{basolateral}}}{M_{\mathrm{initial}}}\times 100$$

where M_apical_, M_cell/membrane_, and M_basolateral_ represent the masses of the compound quantified in each compartment, and M_initial_ represents the nominal mass of the compound applied apically at t = 0 h.

Values represent the percentage (mean ± SD) of the initially applied compound recovered from the apical compartment, the cell-associated/membrane-bound fraction, and the basolateral compartment after 6 h of incubation from three independent biological experiments. Data are reported in Appendix A (Table A2).

#### 4.7.3. Tight Junction Protein Analysis

Intracellular levels of occludin, claudin-1, and ZO-1 were determined in lysates obtained from differentiated Caco-2/HT29-MTX co-cultures using analyte-specific ELISA kits for occludin, claudin-1, and ZO-1 (MyBioSource, San Diego, CA, USA; Cusabio Technology, Houston, TX, USA), according to the manufacturers’ instructions. Absorbance was measured at 450 nm using an Infinite 200 PRO MPlex microplate reader (Tecan, Männedorf, Switzerland). Protein concentrations were calculated from the corresponding standard calibration curves (0–1500 pg/mL for occludin and 0–1000 pg/mL for ZO-1 and claudin-1).

Technical replicates were averaged within each independent experiment before statistical analysis. Results are expressed as percentage change relative to the corresponding untreated control and reported as mean ± SD.

### 4.8. Hepatic Oxidative and Signalling Responses

#### 4.8.1. Superoxide Production

Superoxide production was determined using a cytochrome c reduction assay (Merck Life Science, Rome, Italy), as previously described [90]. Absorbance was measured at 550 nm using an Infinite 200 PRO MPlex microplate reader (Tecan, Männedorf, Switzerland). Superoxide production was calculated as nanomoles of reduced cytochrome c per microgram of total protein.

Technical replicates were averaged within each independent experiment. Results were normalised to the corresponding untreated control and are reported as mean ± SD from five independent biological experiments, each performed in technical triplicate.

#### 4.8.2. ERK1/2 Activation

ERK1/2 activation was assessed in HepG2 cell lysates using the InstantOne™ ELISA kit (Thermo Fisher Scientific, Milan, Italy), which simultaneously quantifies phosphorylated and total ERK1/2 proteins [87]. Briefly, 50 μL of protein lysate prepared in the supplied lysis buffer was added to microplate wells pre-coated with the antibody mixture and incubated for 1 h at room temperature on a plate shaker. Following incubation, the detection reagent was added for 20 min, after which the reaction was terminated with stop solution.

Absorbance was measured at 450 nm using an Infinite 200 PRO MPlex microplate reader (Tecan, Männedorf, Switzerland). ERK1/2 activation was expressed as the ratio of phosphorylated to total ERK1/2, calculated according to the manufacturer’s instructions. Results are expressed as percentage change relative to the untreated control and reported as mean ± SD from five independent biological experiments, each performed in technical triplicate.

### 4.9. Hepatic Cholesterol-Related Pathways

#### 4.9.1. Total and Free Intracellular Cholesterol

Intracellular total and free cholesterol concentrations were determined using a commercial Cholesterol Quantitation Kit (Merck Life Science, Milan, Italy), according to the manufacturer’s instructions [91]. Following treatment, cells were collected, and lipids were extracted using 200 μL of a chloroform:isopropanol:IGEPAL CA-630 mixture (7:11:0.1, *v*/*v*/*v*). After centrifugation at 13,000× *g* for 10 min, the organic phase was collected, and the solvent was evaporated at 50 °C for 30 min. The resulting lipid residue was resuspended in Cholesterol Assay Buffer.

For determination of free cholesterol, samples were incubated with the assay reaction mixture according to the manufacturer’s protocol. Total cholesterol was measured in parallel aliquots, which were incubated with a reaction mixture containing cholesterol esterase to hydrolyse cholesterol esters prior to quantification. Following incubation for 60 min at 37 °C in the dark, absorbance was measured at 570 nm using an Infinite 200 PRO MPlex microplate reader (Tecan, Männedorf, Switzerland). Total and free cholesterol concentrations were calculated from the corresponding standard curves, normalised to total protein content, and expressed as percentage change relative to the corresponding untreated control. Technical replicates were averaged within each independent experiment before statistical analysis. Results are reported as mean ± SD from five independent biological experiments, each performed in technical triplicate.

#### 4.9.2. Total Protein Determination

Total protein concentrations in cell lysates were determined using the bicinchoninic acid protein assay (BCA; Thermo Fisher Scientific, Waltham, MA, USA), according to the manufacturer’s instructions, with bovine serum albumin used as the calibration standard. Absorbance was measured at 562 nm using an Infinite 200 PRO MPlex microplate reader (Tecan, Männedorf, Switzerland). Protein concentrations were used for sample equalisation and normalisation of intracellular assay results.

#### 4.9.3. Intracellular and Extracellular LDL-Associated Cholesterol

LDL-associated cholesterol was quantified in both HepG2 cell lysates and culture supernatants using a colourimetric LDL-C assay based on the cholesterol oxidase–phenol aminophenazone reaction (Elabscience, Wuhan, China), according to the manufacturer’s instructions.

Following treatment, culture supernatants were collected to determine extracellular LDL-associated cholesterol. Prior to lysis, HepG2 cells were extensively washed with PBS to minimise potential contributions from extracellular medium components and loosely cell-associated, serum-derived lipoproteins before intracellular determination. Cells were then harvested and lysed to quantify intracellular LDL-associated cholesterol. According to the analytical principle of the commercial homogeneous assay employed, LDL-associated cholesterol was selectively solubilised and enzymatically quantified, providing a biochemical estimate of LDL-associated cholesterol under the experimental conditions rather than a direct physical quantification of intact LDL particles, receptor occupancy, or intracellular LDL trafficking.

Aliquots of each sample (2.5 μL) were mixed with 180 μL of the supplied Reagent 1 and incubated at 37 °C for 5 min. Absorbance was measured at 546 nm using an Infinite 200 PRO MPlex microplate reader (Tecan, Männedorf, Switzerland).

LDL-associated cholesterol concentrations were calculated from the assay calibration curve. Intracellular values were normalised to total protein content, whereas extracellular values were normalised to the analysed volume of culture supernatant.

Technical replicates were averaged within each independent experiment before statistical analysis. Results are expressed as percentage change relative to the corresponding untreated control and reported as mean ± SD.

#### 4.9.4. LDL Uptake Assay

LDL receptor-mediated uptake was evaluated in HepG2 cells using DiI-labelled low-density lipoprotein (DiI-LDL; 3,3′-dioctadecylindocarbocyanine-labelled LDL) according to a previously described method [92]. To minimise background interference from serum-derived lipoproteins, HepG2 cells were pre-incubated in medium containing lipoprotein-deficient serum (LPDS) prior to the assay. Following treatment, HepG2 cells were incubated with 10 μg/mL DiI-LDL in serum-free DMEM supplemented with 0.5% bovine serum albumin (BSA; Merck Life Science, Rome, Italy) for 4 h at 37 °C to allow receptor-mediated binding and internalisation. To determine non-specific uptake, parallel wells were simultaneously incubated with a 50-fold excess of unlabelled LDL. Following incubation, cells were washed three times with PBS containing 0.2% BSA and twice with PBS alone to remove unbound lipoproteins. Cell-associated DiI fluorescence was extracted with 100% isopropanol for 15 min at room temperature and quantified using an Infinite 200 PRO MPlex microplate reader (Tecan, Männedorf, Switzerland) at excitation and emission wavelengths of 520 and 580 nm, respectively.

Specific DiI-LDL uptake was calculated by subtracting fluorescence measured in the presence of excess unlabelled LDL from total cell-associated fluorescence and was normalised to total protein content. Technical replicates were averaged within each independent experiment before statistical analysis. Results are expressed as percentage change relative to the corresponding untreated control and reported as mean ± SD.

#### 4.9.5. Intracellular Total Bile Acid Levels

Intracellular total bile acid levels were determined in HepG2 cells using a fluorometric assay, as previously described [93]. Following treatment, cells were washed with ice-cold phosphate-buffered saline (PBS), lysed by sonication in cold 1× PBS (Merck Life Science, Milan, Italy), and centrifuged at 10,000× *g* for 10 min at 4 °C. Bile acid levels were quantified from resorufin fluorescence using an Infinite 200 PRO MPlex microplate reader (Tecan, Männedorf, Switzerland) at excitation and emission wavelengths of 560 and 590 nm, respectively. Values were normalised to total protein content.

Technical replicates were averaged within each independent experiment before statistical analysis. Results are expressed as percentage change relative to the corresponding untreated control and reported as mean ± SD.

#### 4.9.6. CYP7A1 Quantification

Intracellular CYP7A1 protein levels were determined using a commercial ELISA kit (FineTest, Wuhan, China) according to the manufacturer’s instructions. Following treatment, HepG2 cells were washed with ice-cold PBS and lysed in RIPA buffer supplemented with protease inhibitors. Cell lysates were centrifuged at 12,000× *g* for 15 min at 4 °C, and the resulting supernatants were collected and adjusted to the same total protein concentration. An aliquot of 100 μL of each sample was added to the antibody-precoated microplate and incubated at 37 °C for 90 min, followed by sequential incubation with a biotin-labelled detection antibody and horseradish peroxidase-conjugated streptavidin. Colour development was achieved using tetramethylbenzidine (TMB) substrate, and absorbance was measured at 450 nm using an Infinite 200 PRO MPlex microplate reader (Tecan, Männedorf, Switzerland).

CYP7A1 concentrations were calculated from the corresponding standard curve (0.156–10 ng/mL). Technical replicates were averaged within each independent experiment before statistical analysis. Results are expressed as percentage change relative to the corresponding untreated control and reported as mean ± SD.

#### 4.9.7. ABCA1 Quantification

Intracellular ABCA1 protein levels were determined using a commercial sandwich ELISA kit for human ABCA1 (Abcam, Cambridge, UK), according to the manufacturer’s instructions. The assay detection range was 0.16–10 ng/mL, with an analytical sensitivity of 0.06 ng/mL. Standards and samples were added to antibody-precoated microplate wells, followed by incubation with the detection antibody and colour development using tetramethylbenzidine (TMB) substrate. Absorbance was measured at 450 nm using an Infinite 200 PRO MPlex microplate reader (Tecan, Männedorf, Switzerland), and ABCA1 concentrations were calculated from the corresponding standard curve. Values were normalised to total protein content. Technical replicates were averaged within each independent experiment before statistical analysis. Results are expressed as percentage change relative to the corresponding untreated control and reported as mean ± SD.

#### 4.9.8. HMGR Quantification

Intracellular HMGR protein levels were determined using a commercial ELISA kit (LSBio, Seattle, WA, USA) according to the manufacturer’s instructions [94]. Following treatment, HepG2 cells were harvested, lysed by three freeze–thaw cycles, and centrifuged at 1500× *g* for 10 min at 2–8 °C. Aliquots of cell lysate (100 μL) were incubated with the biotinylated detection antibody for 60 min at 37 °C, followed by incubation with horseradish peroxidase (HRP)-conjugated streptavidin for 30 min. Colour development was performed using tetramethylbenzidine (TMB) substrate for 15 min in the dark before addition of the stop solution. Absorbance was measured at 450 nm using an Infinite 200 PRO MPlex microplate reader (Tecan, Männedorf, Switzerland). HMGR concentrations were calculated from the corresponding standard curve (0.625–40 ng/mL) and normalised to total protein content. Technical replicates were averaged within each independent experiment before statistical analysis. Results are expressed as percentage change relative to the corresponding untreated control and reported as mean ± SD.

#### 4.9.9. Intracellular and Extracellular PCSK9 Quantification

Intracellular and extracellular PCSK9 levels were determined using commercial ELISA kits from BioVision (Milpitas, CA, USA) and Elabscience Biotechnology (Wuhan, China), respectively, according to the manufacturers’ instructions [95]. Following treatment, culture supernatants were collected for extracellular PCSK9 determination. HepG2 cells were washed with ice-cold phosphate-buffered saline (PBS), lysed in RIPA buffer supplemented with protease inhibitors, and centrifuged at 12,000× *g* for 15 min at 4 °C. The resulting supernatants were collected for intracellular PCSK9 analysis. Absorbance was measured at 450 nm using an Infinite 200 PRO MPlex microplate reader (Tecan, Männedorf, Switzerland). PCSK9 concentrations were calculated from the corresponding standard curves. Intracellular values were normalised to total protein content, whereas extracellular values were expressed relative to the volume of culture medium analysed. Technical replicates were averaged within each independent experiment before statistical analysis. Results are expressed as percentage change relative to the corresponding untreated control and reported as mean ± SD.

#### 4.9.10. SREBP-2 Quantification

Intracellular SREBP-2 protein levels were determined using a commercial ELISA kit (LSBio, Seattle, WA, USA) according to the manufacturer’s instructions. Following treatment, HepG2 cells were lysed according to the kit manufacturer’s instructions, and absorbance was measured at 450 nm using an Infinite 200 PRO MPlex microplate reader (Tecan, Männedorf, Switzerland).

SREBP-2 concentrations were calculated from the corresponding standard curve (0.312–20 ng/mL) and normalised to total protein content. Technical replicates were averaged within each independent experiment before statistical analysis. Results are expressed as percentage change relative to the corresponding untreated control and reported as mean ± SD.

### 4.10. Western Blot Analysis

Following treatment, HepG2 cells were lysed on ice using Radioimmunoprecipitation assay (RIPA) lysis buffer (50 mM Tris-HCl, pH 7.4, 150 mM NaCl, 1% NP-40, 0.5% sodium deoxycholate, and 0.1% sodium dodecyl sulfate, SDS) supplemented with 1 mM phenylmethylsulfonyl fluoride (PMSF), 2 mM sodium orthovanadate, phosphatase inhibitor cocktail (1:50), and protease inhibitor cocktail (1:200) [27]. All reagents indicated previously were purchased from Merck Life Science (Rome, Italy). Equal amounts of protein (35 μg) were separated by 8% SDS-polyacrylamide gel electrophoresis (SDS-PAGE) and transferred onto polyvinylidene fluoride (PVDF) membranes (GE Healthcare Europe GmbH, Milan, Italy). Membranes were incubated overnight at 4 °C with primary antibodies (all diluted 1:500; Santa Cruz Biotechnology, Santa Cruz, CA, USA) against LDLR (mouse monoclonal, Cat. No. sc-18823), phospho-AMPK (Thr172; rabbit polyclonal, Cat. No. sc-33524), and total AMPK (mouse monoclonal, Cat. No. sc-74461). β-Actin (1:4000; Cat. No. A5441, Merck Life Science, Rome, Italy) was used as the loading control. After incubation with horseradish peroxidase-conjugated anti-mouse or anti-rabbit secondary antibodies, as appropriate (1:5000; Merck Life Science, Rome, Italy), immunoreactive bands were visualised by enhanced chemiluminescence according to standard procedures. Band intensities were quantified using Image Lab™ software (version 5.2.1; Bio-Rad, Hercules, CA, USA). LDLR protein expression was normalised to β-actin, whereas AMPK activation was expressed as the phospho-AMPK-to-total AMPK ratio. Representative immunoblots from three independent biological experiments are shown alongside densitometric data from independent biological experiments. At least three independent biological experiments were performed for all Western blot analyses. Representative immunoblots from one biological replicate are shown in the main figures. For each biological replicate, β-actin loading controls were obtained from the same experimental membrane as the corresponding target protein analysis. Densitometric analyses were performed using data from all three independent biological experiments. Results are expressed relative to the corresponding untreated control.

### 4.11. Pharmacological Inhibition of AMPK

To explore the involvement of AMPK-associated signalling, HepG2 cells were pretreated with dorsomorphin (Compound C; 10 μM) for 30 min before exposure to the *Ophiocordyceps sinensis* and CoQ10 combination [68]. Parallel groups treated with dorsomorphin alone were included to distinguish the effects of the inhibitor itself from those of the combined treatment. Following treatment, HMGR, intracellular PCSK9, and LDL uptake were evaluated as described above.

### 4.12. Statistical Analysis

Data are presented as mean ± SD. Unless otherwise specified, five independent biological experiments were performed, with each experimental condition analysed in technical triplicate. Western blot analyses were performed using at least three independent biological experiments, and densitometric analyses were based on data from these independent biological replicates.

Technical replicates were averaged within each independent run to obtain a single value per biological replicate, which was used as the statistical unit (*n*) for all subsequent analyses. This approach was applied consistently to avoid pseudoreplication and ensure that the reported variability reflects biological variation across independent experiments. To determine the appropriate statistical approach, data distribution and homogeneity of variance were assessed using the Shapiro–Wilk and Brown–Forsythe tests, respectively. For datasets satisfying both parametric assumptions (normality and homoscedasticity), comparisons involving a single experimental factor and more than two groups were performed using one-way analysis of variance (ANOVA), followed by Dunnett’s multiple-comparison test, or Tukey’s post hoc test for multiple pairwise comparisons, as specified in the corresponding figure legends. When the assumptions for parametric analysis were not satisfied, non-parametric analysis was applied using the Kruskal–Wallis test followed by Dunn’s multiple-comparison test was used. Comparisons between two independent groups were performed using an unpaired two-tailed Student’s *t*-test or the Mann–Whitney U test, as appropriate.

Pearson correlation analysis was performed to evaluate the linear association between the p-AMPK/AMPK ratio and selected hepatic endpoints. The strength, direction, and significance of the linear associations were described by Pearson’s correlation coefficient (r), coefficient of determination (R^2^), 95% confidence intervals (CI), and exact *p*-values.

TEER and other outcomes evaluated across treatment groups and experimental time points were analysed using two-way ANOVA, with treatment and time as factors, followed by Tukey’s post hoc for multiple-comparisons, as specified in the corresponding figure legends. When measurements were repeatedly obtained from the same experimental insert over time, a two-way repeated-measures ANOVA was used. To ensure maximum statistical transparency, exact *p*-values are reported to three decimal places, where possible, in the text, tables, and figure legends. For highly significant differences, *p*-values are reported as *p* < 0.001. Unless otherwise indicated, data were normalised to the corresponding untreated control and are presented as percentage change relative to control. Statistical analyses were performed using GraphPad Prism version 10.2.3 (GraphPad Software, San Diego, CA, USA). All statistical tests were two-sided, and differences were considered statistically significant at *p* < 0.05.

Potential interactions between *Ophiocordyceps sinensis* and CoQ10 were explored using Bliss independence analysis as an exploratory mathematical assessment of interaction under the specific experimental conditions investigated. Because this analysis was restricted to a single concentration combination, it represents an exploratory screening rather than a definitive pharmacological demonstration of synergy. For outcomes expressed as fractional inhibition (e.g., total cholesterol levels), the expected combined effect was calculated as E_AB_ = E_A_ + E_B_ − E_A_E_B_, where E_A_ and E_B_ represent the effects of the individual treatments normalised to the untreated control. For endpoints expressed as fractional increases relative to the untreated control (e.g., intracellular bile acid levels), the expected additive response was mathematically adapted. For individual fractional responses $E_{A}$ and $E_{B}$, the expected combined response under independent multiplicative action was calculated as $E_{exp}=\left(1+E_{A}\right)\left(1+E_{B}\right)-1$, equivalent to $E_{exp}=E_{A}+E_{B}+\left(E_{A}\times E_{B}\right)$. The observed combined response was then compared to this Bliss-predicted independent-action baseline.

To reflect the experimental uncertainty, the SD of the expected Bliss value was calculated through error propagation using the standard deviations of the individual treatments. No formal statistical comparison was performed between observed and predicted responses; therefore, the analysis is presented as an exploratory assessment of interaction rather than a formal proof of pharmacological synergy. This analysis was exploratory and was not considered a primary statistical endpoint. Cordycepin and CoQ10 concentrations were expressed as absolute values and as percentages of basolateral recovery relative to the amount initially applied to the apical compartment.

## 5. Conclusions

In conclusion, the present study demonstrates that the combined application of *Ophiocordyceps sinensis* extract and CoQ10 results in coordinated changes in selected cholesterol-associated molecular and functional endpoints following sequential intestinal epithelial processing under high-glucose in vitro conditions. The combined treatment-maintained TEER values above the predefined acceptance threshold was associated with measurable basolateral recovery of cordycepin and CoQ10, reduced superoxide production, and modulated multiple components of hepatic cholesterol-related pathways. These responses included changes in HMGR, PCSK9, LDLR, SREBP-2, CYP7A1, ABCA1, LDL uptake, intracellular total bile acids, and AMPK-related signalling. Overall, the findings indicate that the combination modulated multiple interconnected processes involved in cholesterol synthesis, uptake, and cellular handling under high-glucose conditions. These effects do not directly demonstrate improved hepatic cholesterol homeostasis in vivo or enhanced oral bioavailability but suggest that intestinal-processed fractions retain metabolic activity capable of modulating hepatocyte responses under glucose-induced metabolic stress. Within these physiological and methodological boundaries, the sequential in vitro design provides a useful preclinical framework for further mechanistic studies. Future work incorporating direct cholesterol-flux measurements, advanced multicellular or organ-on-chip models, and in vivo validation will be essential to establish causality and assess the translational and therapeutic relevance of the combination.

## Figures and Tables

**Table 1:** Chemical characterisation of *Cordyceps sinensis* extract. Data are expressed as mean ± standard deviation (SD) (*n* = 3).

Natural Extract	Analytical Method	Compound Quantified	Content (% *w*/*w*)
*Cordyceps sinensis*	Phenol–sulfuric acid assay	Total carbohydrates	53.2 ± 2.3
	Congo red assay	β-1,3/1,6-glucans	43.7 ± 1.5
	Aniline Blue assay	β-1,3-glucans	31.8 ± 1.3
	RP-HPLC-UV	Cordycepin	0.47 ± 0.05

## Data Availability

The datasets generated and/or analysed during the current study are included in this published article and its Supplementary Materials. Further information is available from the corresponding author upon reasonable request.

## Supplementary Materials

The following supporting information can be downloaded at https://www.mdpi.com/article/10.3390/molecules31183265/s1, Figure S1. Specific DiI-LDL uptake in HepG2 cells under high-glucose conditions in vitro referred to Table S2. Cells were pre-incubated in lipoprotein-deficient serum (LPDS) and exposed to DiI-LDL in the absence or presence of excess unlabelled LDL. Specific DiI-LDL uptake was calculated by subtracting the fluorescence measured in the presence of excess unlabelled LDL from total cell-associated fluorescence and normalized to total cellular protein. Data are expressed as percentage of Control High Glucose and represent mean ± SD of five independent experiments performed in triplicate. * *p* < 0.05 vs. Control High Glucose; α *p* < 0.05 vs. RYRF; β *p* < 0.05 vs. CoQ10; γ *p* < 0.05 vs. Cordyceps sinensis; Figure S2: Linear correlations between extracellular PCSK9 levels, cellular LDL uptake, and LDLR protein expression. (A) Pearson correlation between extracellular PCSK9 quantification at 24h (% vs. Control) and cellular fluorescent LDL uptake quantification (% vs. Control) (*r* = −0.8878, *R*^2^ = 0.7878, *p* = 0.0002, 95% CI: −0.9550 to −0.7330). (B) Pearson correlation between extracellular PCSK9 quantification at 24h (% vs. Control) and the ratio of LDLR/*β*-actin protein expression (% vs. Control) (*r* = −0.9431, *R*^2^ = 0.8894, *p* < 0.0001, 95% CI: −0.9776 to −0.8591). Solid lines represent the best-fit linear regression; do ed lines denote the 95% confidence intervals. Data points represent individual experimental replicates across conditions. These analyses are interpreted as supportive evidence of biological association and are not presented as proof of causality, which would require dedicated genetic or more selective pharmacological approaches beyond the scope of the present study; Figure S3: Linear correlations between AMPK activation status, cholesterol metabolic markers, and LDL clearance. (A) Pearson correlation between the p-AMPK/AMPK ratio (% vs. Control) and the percentage reduction of HMGR protein levels (*r* = 0.9552, *R*^2^ = 0.9125, 95% CI: 0.8881 to 0.9825, *p* < 0.0001). (B) Pearson correlation between the p AMPK/AMPK ratio (% vs. Control) and extracellular PCSK9 levels quantified in the culture medium at 24h (% vs. Control) (*r* = −0.9370, *R*^2^ = 0.8780, 95% CI: −0.9752 to −0.8447, *p* < 0.0001). (C) Pearson correlation between the p AMPK/AMPK ratio (% vs. Control) and cellular fluorescent LDL uptake quantification (% vs. Control) (*r* = 0.9911, *R*^2^ = 0.9823, 95% CI: 0.9771 to 0.9966, *p* < 0.0001). Solid lines represent the best-fit linear regression; do ed lines denote the 95% confidence intervals. Data points represent individual experimental replicates across conditions. These analyses are interpreted as supportive evidence of biological association and are not presented as proof of causality, which would require dedicated genetic or more selective pharmacological approaches beyond the scope of the present study; Table S1: Effects of physiological glucose, osmotic control, and high-glucose conditions on functional endpoints in HepG2 cells in vitro. Cells were exposed to 5 mM glucose, 5 mM glucose + 24.5 mM mannitol, or 30 mM glucose. Values are expressed as mean ± SD of independent experiments performed in triplicate. *p*-values refer to the comparison between physiological glucose and osmotic control; Table S2: Receptor-specific DiI-LDL uptake in HepG2 cells under high-glucose conditions in vitro. Total DiI-LDL uptake was measured in the absence or presence of excess unlabelled LDL, and specific uptake was calculated by subtracting the competitively inhibited fraction from total cell-associated fluorescence. Values are expressed as mean ± SD of independent experiments performed in triplicate. Percentage change indicates the difference versus Control High Glucose; Table S3: Analytical validation parameters of the RP-HPLC-UV method for cordycepin quantification in biological samples.

## Abbreviations

The following abbreviations are used in this manuscript:

ABCA1	ATP-binding cassette transporter A1
AMPK	AMP-activated protein kinase
ANOVA	Analysis of variance
ATCC	American Type Culture Collection
Caco-2	Human colorectal adenocarcinoma epithelial cell line
CoQ10	Coenzyme Q10 (ubiquinone)
CYP7A1	Cytochrome P450 family 7 subfamily A member 1
DMEM	Dulbecco’s Modified Eagle Medium
DMSO	Dimethyl sulfoxide
ERK1/2	Extracellular signal-regulated kinase 1/2
FBS	Foetal bovine serum
HMGR	3-Hydroxy-3-methylglutaryl-CoA reductase
HPLC-UV	High-performance liquid chromatography coupled with ultraviolet–visible
HT29-MTX	Methotrexate-adapted human colorectal adenocarcinoma mucus-producing cell line
LDL	Low-density lipoprotein
LDLR	Low-density lipoprotein receptor
MAPK	Mitogen-activated protein kinase
MLCK	Myosin light chain kinase
MTT	3-(4,5-Dimethylthiazol-2-yl)-2,5-diphenyltetrazolium bromide
PBS	Phosphate-buffered saline
PCSK9	Proprotein convertase subtilisin/kexin type 9
PMSF	Phenylmethylsulfonyl fluoride
PVDF	Polyvinylidene fluoride
RIPA	Radioimmunoprecipitation assay buffer
ROS	Reactive oxygen species
RP-HPLC-UV	Reverse-phase high-performance liquid chromatography coupled with ultraviolet detection
RYRF	Red yeast rice formulation
SREBP-2	Sterol regulatory element-binding protein 2
TEER	Transepithelial electrical resistance
TJ	Tight junction

## Appendix A

**Table A1:** Basolateral transport of cordycepin and CoQ10 in a Caco-2/HT29-MTX Transwell^®^ model under single (Alone) and combined (MIX) treatment conditions. Cordycepin and CoQ10 concentrations were quantified by HPLC in the basolateral compartment after 6 h of exposure. Values are expressed as mean ± SD (*n* = 3). Data reflect the fraction of compounds crossing the Caco-2/HT29-MTX co-cultures from the apical to the basolateral side. Initial apical concentrations were 0.94 µg/mL for cordycepin (from a *Cordyceps sinensis* 200 µg/mL extract, 0.47 ± 0.05% *w*/*w*) and 43 µg/mL for CoQ10.

Sample	Cell Model	Time (h)	Condition	Basolateral Concentration (µg/mL)	% of Initial Dose
Cordycepin (*Cordyceps sinensis*)	Caco-2/HT29-MTX (Transwell^®^)	6	Alone	0.27 ± 0.04	28.7 ± 4.6
			MIX	0.34 ± 0.05	36.2 ± 5.3
CoQ10			Alone	1.12 ± 0.18	2.6 ± 0.4
			MIX	1.58 ± 0.26	3.7 ± 0.6

**Table A2:** Mass-balance analysis and analytical recovery of cordycepin and coenzyme Q10 across differentiated Caco-2/HT29-MTX monolayers following 6 h of apical exposure. Values represent the percentage (mean ± SD) of the initially applied compound recovered from the apical compartment, the cell-associated/membrane-bound fraction, and the basolateral compartment after 6 h of incubation from three independent biological experiments (*n* = 3). Total mass-balance recovery corresponds to the sum of these recovered fractions and was used to assess the overall analytical recovery of the experimental system and the proportion of compound remaining unaccounted for. Data are shown for cordycepin, CoQ10, and their combined treatment.

Sample	Apical Remaining (%)	Cell-Associated/Membrane-Bound (%)	Basolateral Recovered (%)	Total Mass Balance Recovery (%)
Cordycepin (*Cordyceps sinensis*)	81.2 ± 3.4	3.5 ± 0.4	7.8 ± 0.6	92.5 ± 3.8
CoQ10	45.2 ± 4.1	18.6 ± 2.2	4.5 ± 0.5	68.3 ± 4.5
*Cordyceps sinensis* + CoQ10	58.4 ± 3.8	21.5 ± 1.8	5.8 ± 0.5	85.7 ± 4.1

**Table A3:** Bliss independence analysis on total cholesterol. The observed response represents a greater-than-additive effect under the Bliss model without formal statistical comparison between observed and predicted values.

Treatment	Expected Data	Observed Data	Interaction
*Cordyceps sinensis* + CoQ10	−46.95 ± 3.12%	−54.10 ± 2.90%	Greater-than-additive (Bliss)

**Table A4:** Bliss independence analysis on intracellular total bile acid content. The observed response represents a greater-than-additive effect under the Bliss model without formal statistical comparison between observed and predicted values.

Treatment	Expected Data	Observed Data	Interaction
*Cordyceps sinensis* + CoQ10	27.85 ± 2.15%	33.00 ± 1.85%	Greater-than-additive (Bliss)

**Table A5:** Extracellular recovery and persistence of intact cordycepin and CoQ10 in the HepG2 culture medium following 24 h exposure. Data represent the fraction of cordycepin and CoQ10 detectable in the extracellular supernatant of HepG2 compartment after 24 h of exposure to post-intestinal basolateral fractions. Values are expressed as mean ± SD (*n* = 3). Initial apical concentrations were 0.94 µg/mL for cordycepin (from a *Cordyceps sinensis* 200 µg/mL extract, 0.47 ± 0.05% *w*/*w*) and 43 µg/mL for CoQ10.

Sample	Cell Model	Time (h)	Condition	Detected Concentration (µg/mL)	% of Initial Dose
Cordycepin (*Cordyceps sinensis*)	HepG2 (Transwell^®^)	24 h	Alone	0.14 ± 0.03	14.9 ± 3.2
			MIX	0.19 ± 0.04	20.2 ± 4.1
CoQ10			Alone	0.62 ± 0.14	1.4 ± 0.3
			MIX	0.88 ± 0.17	2.0 ± 0.4

## Appendix B

**Table A6:** Effects of different treatments on transepithelial electrical resistance (TEER) in Caco-2/HT29 cells in vitro. TEER values (Ω·cm^2^) were measured at 1–6 h following treatment. Values are expressed as mean ± SD of 5 experiments performed in triplicate. Control represents untreated cells under the corresponding experimental conditions.

Time	Control	RYRF 8 μg/mL	CoQ10 43 μg/mL	*Cordyceps* *sinensis* 200 μg/mL	*Cordyceps* *sinensis* + CoQ10
1 h	410.5 ± 0.71	420.0 ± 1.76	471.5 ± 2.12	426.0 ± 1.41	491.5 ± 2.21
2 h	410.5 ± 0.93	426.0 ± 1.41	489.5 ± 3.54	446.0 ± 1.48	514.0 ± 2.83
3 h	412.5 ± 0.78	427.5 ± 2.19	494.0 ± 4.24	450.5 ± 2.12	520.5 ± 2.17
4 h	411.5 ± 0.99	424.0 ± 1.41	502.5 ± 3.54	449.5 ± 2.08	525.5 ± 0.72
5 h	410.0 ± 1.41	419.5 ± 0.99	494.5 ± 3.71	443.0 ± 2.83	520.0 ± 4.24
6 h	410.5 ± 0.71	416.5 ± 0.77	490.5 ± 2.12	439.0 ± 1.55	510.5 ± 2.12

**Table A7:** Effects of different treatments on total cholesterol levels in HepG2 cells under high-glucose conditions in vitro. Raw data (µg/µL protein) are associated with the graph shown in Figure 4B. Values are expressed as mean ± SD of independent experiments performed in triplicate. Percentage change indicates the difference versus Control Normal Glucose.

Samples	Free Cholesterol (µg/µL) Mean ± SD	% Change vs. Control High Glucose
Control Normal Glucose	3.76 ± 0.13	90.00
Control High Glucose	1.98 ± 0.07	
RYRF	3.36 ± 0.05	69.75
CoQ10	3.31 ± 0.04	67.50
*Cordyceps sinensis*	4.31 ± 0.08	117.60
*Cordyceps sinensis* + CoQ10	4.62 ± 0.05	133.50

**Table A8:** Effects of different treatments on DiI-LDL uptake in HepG2 cells under high-glucose conditions in vitro. Raw data are expressed as relative fluorescence units per microgram of protein and (RFU/µg protein) are associated with the graph shown in Figure 4E. Values are expressed as mean ± SD of independent experiments performed in triplicate. Percentage change indicates the difference versus Control High Glucose.

Samples	DiI-LDL Uptake (RFU/Protein) Mean ± SD	% Change vs. Control High Glucose
Control High Glucose	987 ± 44	
RYRF	1022 ± 46	3.50
CoQ10	1039 ± 47	5.30
*Cordyceps sinensis*	1101 ± 50	11.50
*Cordyceps sinensis* + CoQ10	1245 ± 56	26.14

**Table A9:** Effects of different treatments on intracellular total bile acid levels in HepG2 cells under high-glucose conditions in vitro. Raw data (µg/µL protein) are associated with the graph shown in Figure 5A. Values are expressed as mean ± SD of independent experiments performed in triplicate. Percentage change indicates the difference versus Control High Glucose.

Samples	Bile Acid (µg/µL Protein) Mean ± SD	% Change vs. Control High Glucose
Control High Glucose	0.675 ± 0.05	
RYRF	0.709 ± 0.06	5.00
CoQ10	0.726 ± 0.06	7.50
*Cordyceps sinensis*	0.823 ± 0.08	21.87
*Cordyceps sinensis* + CoQ10	0.898 ± 0.09	33.06

**Table A10:** Effects of different treatments on free cholesterol levels in HepG2 cells under high-glucose conditions in vitro. Raw data (µg/µL protein) are associated with the graph shown in Figure 5C. Values are expressed as mean ± SD of independent experiments performed in triplicate. Percentage change indicates the difference versus Control High Glucose.

Samples	Total Cholesterol (µg/µL) Mean ± SD	% Change vs. Control Normal Glucose
Control Normal Glucose	2.97 ± 0.12	
Control High Glucose	5.05 ± 0.18	70.00
RYRF	3.46 ± 0.13	16.01
CoQ10	3.48 ± 0.14	17.46
*Cordyceps sinensis*	1.92 ± 0.09	−35.72
*Cordyceps sinensis* + CoQ10	1.36 ± 0.09	−54.08

**Table A11:** Effects of different treatments on extracellular PCSK9 levels in HepG2 cells under high-glucose conditions in vitro. Raw data (pg/µL protein) are associated with the graph shown in Figure 6A. Values are expressed as mean ± SD of independent experiments performed in triplicate. Percentage change indicates the difference versus Control High Glucose.

Samples	PCSK9 Intra (pg/mL) Mean ± SD	% Change vs. Control High Glucose
Control High Glucose	287.12 ± 7.23	
RYRF	272.04 ± 6.32	−5.25
CoQ10	273.05 ± 6.38	−4.90
*Cordyceps sinensis*	208.51 ± 12.51	−27.37
*Cordyceps sinensis* + CoQ10	162.29 ± 9.74	−43.46

**Table A12:** Effects of different treatments on intracellular PCSK9 levels in HepG2 cells under high-glucose conditions in vitro. Raw data (pg/µL protein) are associated with the graph shown in Figure 6B. Values are expressed as mean ± SD of independent experiments performed in triplicate. Percentage change indicates the difference versus Control High Glucose.

Samples	PCSK9 Extra (pg/mL) Mean ± SD	% Change vs. Control High Glucose
Control High Glucose	4.96 ± 0.32	
RYRF	5.65 ± 0.36	14.00
CoQ10	4.56 ± 0.29	−8.00
*Cordyceps sinensis*	1.97 ± 0.13	−60.40
*Cordyceps sinensis* + CoQ10	1.31 ± 0.08	−73.50
