# Supplementary material for: Modulation of Hepatic Cholesterol-Related Pathways by Ophiocordyceps sinensis and Coenzyme Q10 in a Sequential Gut–Liver In Vitro Model
Source: Molecules. 2026 Sep 15;31(18):3265. doi: 10.3390/molecules31183265 (PMC13609694; doi:10.3390/molecules31183265)
Supplement: Supplementary file 1 [file molecules-31-03265-s001.zip › molecules-4502142-supplementary.pdf]

Supplementary Materials

Table S1. Effects of physiological glucose, osmotic control, and high-glucose conditions on functional endpoints in HepG2 cells in vitro. Cells were exposed to 5 mM glucose, 5 mM glucose + 24.5 mM mannitol, or 30 mM glucose. Values are expressed as mean ± SD of independent experiments performed in triplicate. p-values refer to the comparison between physiological glucose and osmotic control.

Parameter	5 mM Glucose	5 mM + 24.5 mM Mannitol	30 mM Glucose	*p*-value
**Total cholesterol (μg/μL protein)**	2.97 ± 0.12	2.94 ± 0.14	5.05 ± 0.18	0.69
**Intracellular Bile acid levels (μg/μL protein)**	0.810 ± 0.08	0.798 ± 0.07	0.675 ± 0.05	0.81
**DiI-LDL uptake (RFU/μg protein)**	1087 ± 32	1074 ± 35	987 ± 44	0.56
**Extracellular PCSK9 (pg/mL)**	3.06 ± 0.17	3.11 ± 0.16	4.96 ± 0.32	0.64
**Intracellular PCSK9 (pg/mL)**	227.12 ± 8.53	230.46 ± 9.12	287.12 ± 7.23	0.57

Table S2. Receptor-specific DiI-LDL uptake in HepG2 cells under high-glucose conditions in vitro. Total DiI-LDL uptake was measured in the absence or presence of excess unlabelled LDL, and specific uptake was calculated by subtracting the competitively inhibited fraction from total cell-associated fluorescence. Values are expressed as mean ± SD of independent experiments performed in triplicate. Percentage change indicates the difference versus Control High Glucose.

Treatment	Total DiI-LDL uptake (RFU/μg protein) Mean ± SD	Competition control (RFU/μg protein) Mean ± SD	Specific DiI-LDL uptake (RFU/μg protein) Mean ± SD	Specific uptake (% vs Control High Glucose)
**Control High Glucose**	987 ± 44	175 ± 18	812 ± 40	
**RYRF**	1,022 ± 46	181 ± 19	841 ± 42	3.57
**CoQ10**	1,039 ± 47	184 ± 19	855 ± 43	5.30
Cordyceps sinensis	1,101 ± 50	190 ± 20	911 ± 45	12.19
**Cordyceps sinensis + CoQ10**	1,245 ± 56	205 ± 21	1,040 ± 50	28.08

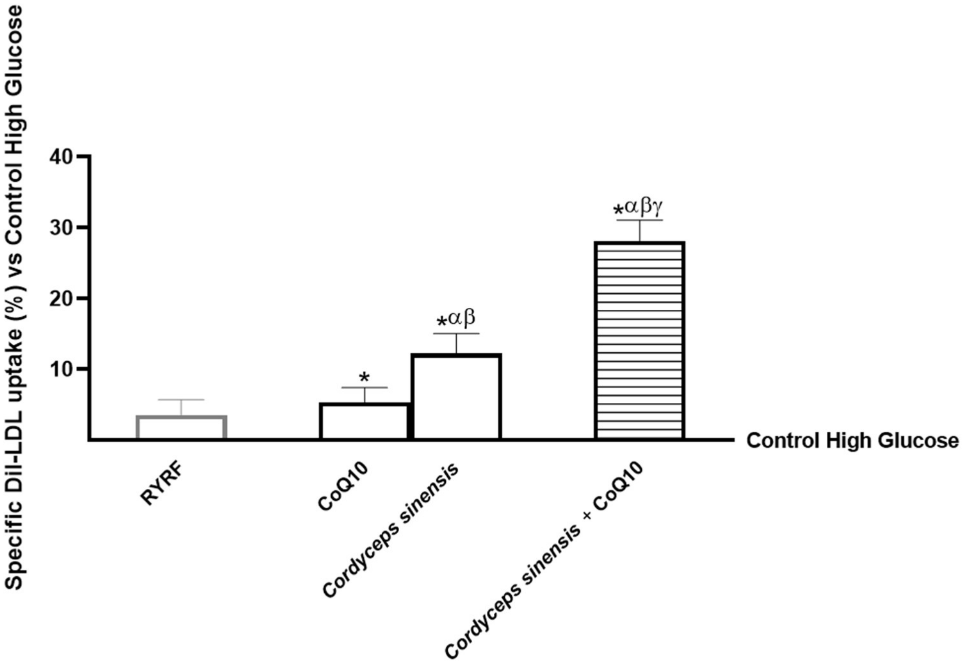

Figure S1. Specific DiI-LDL uptake in HepG2 cells under high-glucose conditions in vitro referred to Table S2. Cells were pre-incubated in lipoprotein-deficient serum (LPDS) and exposed to DiI-LDL in the absence or presence of excess unlabelled LDL. Specific DiI-LDL uptake was calculated by subtracting the fluorescence measured in the presence of excess unlabelled LDL from total cell-associated fluorescence and normalized to total cellular protein. Data are expressed as percentage of Control High Glucose and represent mean ± SD of five independent experiments performed in triplicate. * p < 0.05 vs. Control High Glucose; α p < 0.05 vs. RYRF; β p < 0.05 vs. CoQ10; γ p < 0.05 vs. *Cordyceps sinensis*.

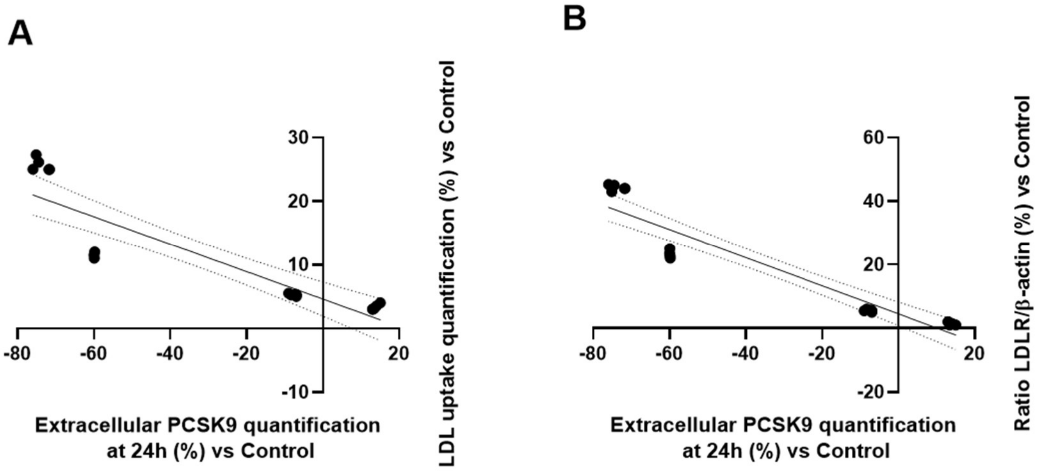

Figure S2. Linear correlations between extracellular PCSK9 levels, cellular LDL uptake, and LDLR protein expression. (A) Pearson correlation between extracellular PCSK9 quantification at 24h (% vs. Control) and cellular fluorescent LDL uptake quantification (% vs. Control) ($r = -0.8878, R^2 = 0.7878, p = 0.0002$, 95% CI: -0.9550 to -0.7330). (B) Pearson correlation between extracellular PCSK9 quantification at 24h (% vs. Control) and the ratio of LDLR/β-actin protein expression (% vs. Control) ($r = -0.9431$, $R^2 = 0.8894$, $p < 0.0001$, 95% CI: -0.9776 to -0.8591). Solid lines represent the best-fit linear regression; dotted lines denote the 95% confidence intervals. Data points represent individual experimental replicates across conditions. These analyses are interpreted as supportive evidence of biological association and are not presented as proof of causality, which would require dedicated genetic or more selective pharmacological approaches beyond the scope of the present study.

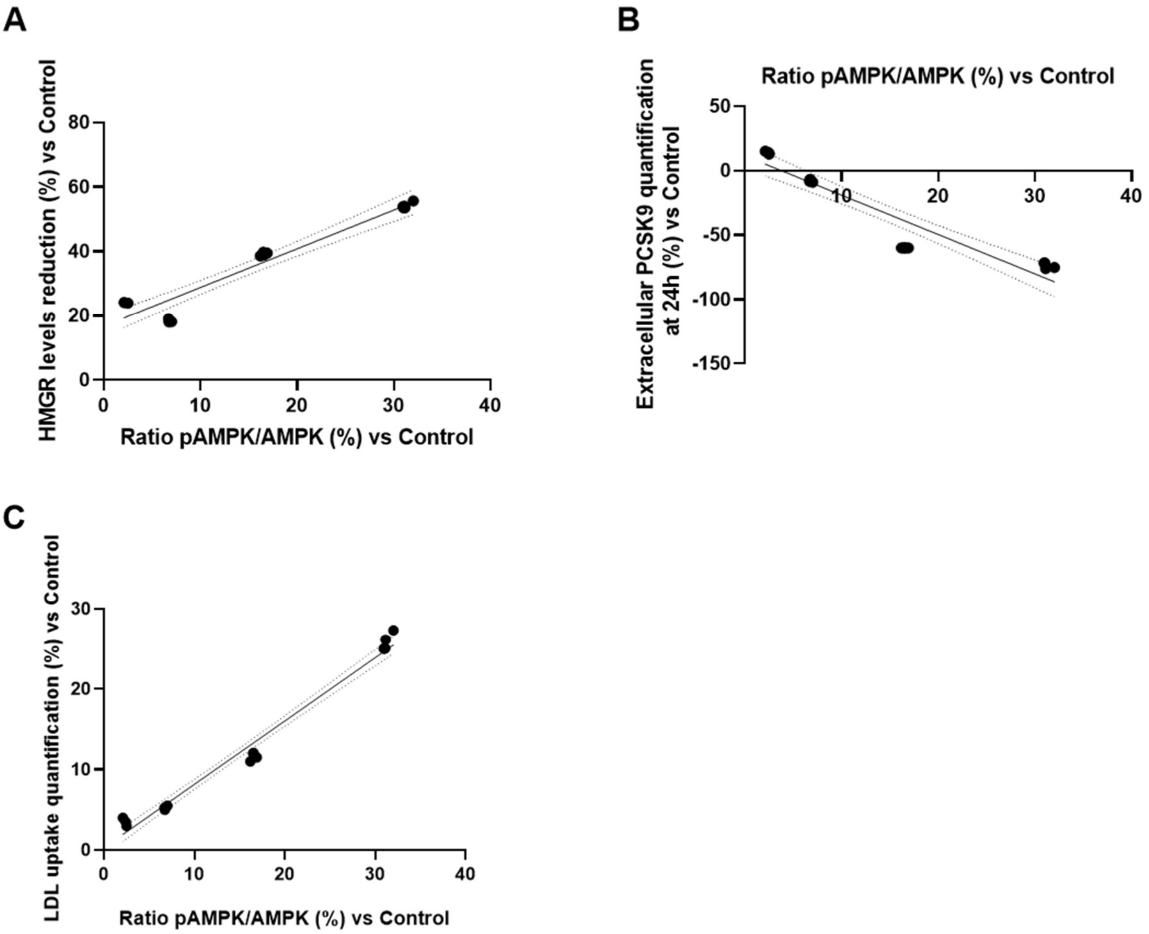

Figure S3. Linear correlations between AMPK activation status, cholesterol metabolic markers, and LDL clearance. (A) Pearson correlation between the p-AMPK/AMPK ratio (% vs. Control) and the percentage reduction of HMGR protein levels ($r = 0.9552,\ R^2 = 0.9125,\ 95\%\ \text{CI}: 0.8881\text{ to }0.9825,\ p < 0.0001$). (B) Pearson correlation between the p-AMPK/AMPK ratio (% vs. Control) and extracellular PCSK9 levels quantified in the culture medium at 24h (% vs. Control) ($r = -0.9370,\ R^2 = 0.8780,\ 95\%\ \text{CI}: -0.9752\text{ to }-0.8447,\ p < 0.0001$). (C) Pearson correlation between the p-AMPK/AMPK ratio (% vs. Control) and cellular fluorescent LDL uptake quantification (% vs. Control) ($r = 0.9911$, $R^2 = 0.9823$, 95% CI: 0.9771 to 0.9966, $p < 0.0001$). Solid lines represent the best-fit linear regression; dotted lines denote the 95% confidence intervals. Data points represent individual experimental replicates across conditions. These analyses are interpreted as supportive evidence of biological association and are not presented as proof of causality, which would require dedicated genetic or more selective pharmacological approaches beyond the scope of the present study.

Table S3. Analytical validation parameters of the RP-HPLC-UV method for cordycepin quantification in biological samples.

Parameter	Value
**Calibration range**	0.05–2.00 µg/mL
**Linearity (R^2)**	0.9994
**LOD**	0.015 µg/mL
**LOQ**	0.050 µg/mL
**Recovery**	91.5 ± 3.4%
**Intra-day precision**	3.8%
**Inter-day precision**	5.2%
**Retention time**	8.9 ± 0.1 min
